# DNA Methylation and Chromatin Accessibility Impact Subgenome Expression Dominance in the Common Carp (*Cyprinus carpio*)

**DOI:** 10.3390/ijms25031635

**Published:** 2024-01-29

**Authors:** Shuang-Ting Yu, Ran Zhao, Xiao-Qing Sun, Ming-Xi Hou, Yi-Ming Cao, Jin Zhang, Ying-Jie Chen, Kai-Kuo Wang, Yan Zhang, Jiong-Tang Li, Qi Wang

**Affiliations:** 1 Key Laboratory of Aquatic Genomics, Ministry of Agriculture and Rural Affairs and Beijing Key Laboratory of Fishery Biotechnology, Chinese Academy of Fishery Sciences, Beijing 100141, China; styuwork@163.com (S.-T.Y.); zhaoran@cafs.ac.cn (R.Z.); sunxiaoqing@cafs.ac.cn (X.-Q.S.); houmingxi@cafs.ac.cn (M.-X.H.); caoyiming@cafs.ac.cn (Y.-M.C.); zhangjin@cafs.ac.cn (J.Z.); cyjttkl@163.com (Y.-J.C.); 18631836881@163.com (K.-K.W.); zhangyan@cafs.ac.cn (Y.Z.); 2 Chinese Academy of Agricultural Sciences, Beijing 100081, China

**Keywords:** polyploidy, subgenome expression, DNA methylation, chromatin accessibility, homoeologous gene

## Abstract

DNA methylation and chromatin accessibility play important roles in gene expression, but their function in subgenome expression dominance remains largely unknown. We conducted comprehensive analyses of the transcriptome, DNA methylation, and chromatin accessibility in liver and muscle tissues of allotetraploid common carp, aiming to reveal the function of epigenetic modifications in subgenome expression dominance. A noteworthy overlap in differential expressed genes (DEGs) as well as their functions was observed across the two subgenomes. In the promoter and gene body, the DNA methylation level of the B subgenome was significantly different than that of the A subgenome. Nevertheless, differences in DNA methylation did not align with changes in homoeologous biased expression across liver and muscle tissues. Moreover, the B subgenome exhibited a higher prevalence of open chromatin regions and greater chromatin accessibility, in comparison to the A subgenome. The expression levels of genes located proximally to open chromatin regions were significantly higher than others. Genes with higher chromatin accessibility in the B subgenome exhibited significantly elevated expression levels compared to the A subgenome. Contrastingly, genes without accessibility exhibited similar expression levels in both subgenomes. This study contributes to understanding the regulation of subgenome expression dominance in allotetraploid common carp.

## 1. Introduction

As multiple subgenomes coexist in the same cell, one subgenome exhibits prevalent higher levels of gene expression, a phenomenon known as subgenome expression dominance. Subgenome expression dominance is considered an important driver for resolving genomic incompatibility [1] and shaping environmental adaptability [2]. Early in the generation of allotetraploids or hybrids, subgenome expression dominance was established and subsequently enhanced in successive generations [3,4]. Most allotetraploids without genetic differences do not exhibit subgenome dominance [5], and reproducible and predictable subgenome dominance patterns have been observed in synthesized allopolyploids [6]. However, this is not a static situation. The dominant subgenome can be switched at specific developmental stages or in response to stimuli [5,7], suggesting the dynamic nature of inter-subgenome expression patterns. However, little is known about the regulation of subgenome expression dominance.

Discrepancies in transposable element (TE) contents, along with associated DNA methylation patterns, are considered significant contributors to subgenome expression dominance. In allotetraploids and interspecific hybrids, subgenomes with lower transposon content usually have higher expression levels [5], whereas autotetraploids with comparable transposon loads usually exhibit balanced expression levels [8]. In most cases, however, inactive transposons cannot directly regulate gene expression but instead influence nearby gene expression through DNA methylation. As a reversible epigenetic modification, DNA methylation is primarily enriched in the TE region and functions to inhibit TE activity [9]. DNA methylation plays an important role in the regulation of gene expression, and DNA methylation in promoters is the most common epigenetic modification that suppresses gene expression. Therefore, DNA methylation emerges as the primary candidate for the regulation of subgenome expression dominance. In allopolyploids, the decrease in gene expression level in a specific subgenome is accompanied by an increased DNA methylation level [5]. A study of DNA methylation and gene expression in resynthesized allopolyploids provided further evidence of this [6]. In F1 hybrids, one subgenome presents significantly higher DNA methylation levels and lower gene expression levels than the other. Further enhancements in DNA methylation level differentiation are followed by the maintenance of subgenome dominance in subsequent generations. 

Transcription is controlled by interactions between regulatory proteins and DNA regulatory elements. Chromatin accessibility, the degree to which proteins can contact chromatinized DNA, plays a crucial role in gene expression regulation [10]. Assay for transposase-accessible chromatin using sequencing (ATAC-seq) is a simple and highly reproducible method for discerning open chromatin regions [11]. Recently, ATAC-seq technology has been applied to identify DNA regulatory elements involved in liver and gonadal development [12,13]. In polyploids, ATAC-seq has also been used to analyze genome-wide differential chromatin accessibility landscapes. Compared to those in diploid ancestors, large-scale changes in chromatin accessibility associated with gene expression have been observed in allopolyploids [14,15]. Nevertheless, the relationship between dynamic chromatin accessibility and subgenome expression dominance has still largely been unexplored. 

The common carp (*Cyprinus carpio*) is one of the most important cultured fish in China and a model organism representing the *Cyprinidae* family. As a rare polyploid animal, the common carp probably evolved from interspecific hybridization 13.5~25.6  million years ago [16]. Two sets of genetic material from two parents, A and B subgenomes, have been identified in the common carp. The two subgenomes maintain similar chromosome structures, including equivalent gene and transposon contents. A higher gene expression and stronger stress responses have been identified in the B subgenome [17]. DNA methylation is thought to be responsible for homoeologous biased expression in muscle tissue [17]. However, how DNA methylation regulates subgenome expression dominance in different tissues is currently poorly understood. In addition, the role of chromatin accessibility in subgenome dominance has not been determined in the common carp. Therefore, we performed a comprehensive analysis of gene expression, DNA methylation, and chromatin accessibility in the liver and muscle tissues of the common carp to investigate the regulation of subgenome dominance in the different tissues. This study provides valuable insights into the epigenetic basis of subgenome dominance.

## 2. Results

### 2.1. Biased Differential Expression and Function between the Two Subgenomes

In total, 130 million clean RNA-seq reads were generated for liver and muscle tissues, yielding an average of 21.67 million reads per sample (Supplementary Table S1). Approximately 80% of the clean reads were uniquely mapped to the reference genome, and the overall mapping ratio was greater than 93%. PCA analysis of gene expression levels revealed that the first principal component (PC1) and the second principal component (PC2) explained 76.7% and 7.8%, respectively, of the total variance. A clear distinction could be made between the liver and muscle tissue in PC1, and a high degree of similarity among the three biological replicates was observed (Supplementary Figure S1A). Saturation curves for each tissue showed that the number of expressed genes approached saturation at roughly half of the sequence reads (Supplementary Figure S1B). 

A total of 65.64% and 51.56% of genes originating from the A subgenome were expressed in liver and muscle tissue, respectively, which was significantly lower than the expression of the B subgenome (68.13% and 53.62%, χ^2^ test, *p* = 8.60 × 10^−10^ and 1.71 × 10^−6^, Supplementary Figure S2). Among 16,472 homoeologous pairs, 76.08% and 64.08% homoeologs in the A subgenome were detected to be expressed in liver and muscle tissue, respectively, which was also lower than the proportion of expressed homoeologs originating from the B subgenome (78.03% and 66.62%, χ^2^ test, *p* = 6.00 × 10^−5^ and 9.45 × 10^−5^, Supplementary Figure S2). There was also a significant increase in gene expression levels in the B subgenome (Supplementary Figure S3). 

**Figure 1:**
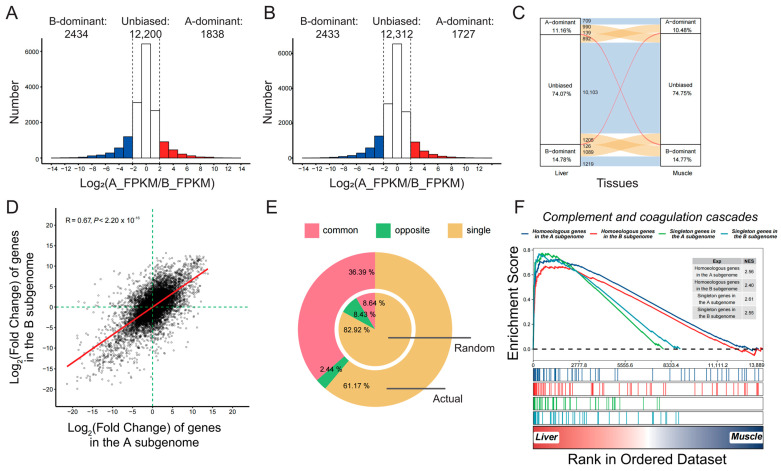
Biased differential expression and function between the two subgenomes. Changes in the expression of homoeologs in the liver (**A**) and muscle (**B**). (**C**) Changes in gene expression change categories in different tissues. Yellow and red alluvial flow represent homoeologs that change expression categorization. (**D**) Correlations between fold changes in homoeologous genes expression levels in the A and B subgenomes. (**E**) The proportion of homoeologous pairs with differential expression patterns between actual and random data. (**F**) Gene set enrichment analysis (GSEA) of homoeologous and singleton genes in the A and B subgenomes. NES represents normalized enrichment score.

By comparing homoeolog expression levels between the two subgenomes, we found that there were three gene expression categories: A-dominant, B-dominant, and unbiased expression (Figure 1A,B). The number of homoeologous pairs with unbiased expression was the highest, with 12,200 in the liver and 12,312 in the muscle, respectively. The number of homoeologous pairs with B-dominant expression (2424 in the liver and 2433 in the muscle) was always greater than that of pairs with A-dominant expression (1838 in the liver and 1727 in the muscle, χ^2^ test, *p* < 2.20 × 10^−16^). Most homoeologous pairs (11,901, 72.25%) did not change their expression types in different tissues, and only 265 pairs (1.61%) switched between A-dominant and B-dominant expression (Figure 1C). Pairs with B-dominant expression in both tissues accounted for 32.27% of pairs with B-dominant expression in any tissue, which was significantly greater than that of pairs with A-dominant expression (24.82%, χ^2^ test, *p* = 4.65 × 10^−11^). Similarly, the number of homoeologous pairs shifted in A-dominant and unbiased expression (65.90%) was significantly greater than that in B-dominant and unbiased expression (60.71%, χ^2^ test, *p* = 1.73 × 10^−05^). The expression of singleton genes was also compared between the two subgenomes, and a significant increase in the proportion and abundance of singleton genes expressed in liver and muscle tissue was observed in the B subgenome (Supplementary Figures S2 and S3). Interestingly, the expression levels of homoeologous genes were almost two times higher than those of singletons (Supplementary Figure S3).

A total of 14,436 differentially expressed genes (DEGs) were identified between the liver and muscle (Supplementary Figure S4). There were 8470 genes with significantly higher expression levels in the liver than in muscle tissue, and these genes were enriched in 2606 GO terms (Supplementary Table S2). Many GO terms involved in responses to stimuli and immune processes, such as leukocyte activation (GO:0045321), NK T cell activation (GO:0051132), respiratory bursts (GO:0045730), and the immune response-regulating signaling pathway (GO:0002764), were enriched. In addition, these genes were also associated with small-molecule biosynthetic processes (GO:0044283), including amino acid, lipid, glycoside, and hormone metabolism, and secretion by cells (GO:0032940), including vitamin, xenobiotic, and organic acid transport. A total of 5966 genes highly expressed in muscle tissue were enriched in 1460 GO terms (Supplementary Table S3), including muscle contraction (GO:0006936), actin filament-based movement (GO:0030048), and mitochondrial respiratory chain complex assembly (GO:0033108). Substance metabolism in the muscle was dominated by glycolytic fermentation (GO:0019660) and nucleoside phosphate metabolic processes (GO:0006753). These DEGs were closely related to tissue differentiation, wherein the liver serves as a primary metabolic organ and immune regulator [18], whereas muscle tissue is the main source of power enabling the fish to swim [19].

There were 2440 and 2048 differentially expressed homoeologous genes in the B subgenome in the liver and muscle, respectively, compared with 2365 and 1866 genes in the A subgenome. The proportion of homoeologous genes highly expressed in muscle tissue was significantly greater in the B subgenome (13.59%) than in the A subgenome (12.52%, χ^2^ test, *p* = 6.31 × 10^−3^). However, there was no difference in the liver (15.87% vs. 16.19%, χ^2^ test, *p* = 0.46). To investigate the contribution of DEGs to tissue differentiation, gene expression changes were analyzed. There was a significant positive correlation between the fold changes of homologs across both subgenomes (Figure 1D). Moreover, 36.39% of homoeologous pairs were highly expressed in the same tissue. The proportion was significantly greater than random (8.64%, χ^2^ test, *p* < 2.2 × 10^−16^, Figure 1E). The number of homoeologous pairs with opposite expression patterns (2.44%) was significantly lower than expected (8.43%, χ^2^ test, *p* < 2.2 × 10^−16^). Interestingly, the percentage of pairs with only one DEG (61.17%) was also significantly lower than random (82.92%, χ^2^ test, *p* < 2.2 × 10^−16^). On the other hand, the proportion of differentially expressed singleton genes in the B subgenome was greater than in the A subgenome but was significantly lower than that of homoeologous genes (Supplementary Figure S5).

In the A and B subgenomes, 719 and 550 GO terms were significantly enriched for highly expressed homoeologous genes in the liver, respectively (Supplementary Figure S6). A total of 375 GO terms were shared by both groups (Supplementary Table S4), including leukocyte activation (GO:0045321), respiratory bursts (GO:0045730), the immune response (GO:0006955), and small-molecule biosynthetic processes (GO:0044283). Several GO terms were specifically enriched in the B subgenome, including hemopoiesis (GO:0030097), signal transduction (GO:0007165), epithelium development (GO:0060429), and mitotic DNA replication (GO:1902969). The GO terms specific to the A subgenome focused on lymphocyte mediated immunity (GO:0002449), regulation of neutrophil migration (GO:1902622), peptide hormone secretion (GO:0030072), and lipid homeostasis (GO:0055088). Singleton genes highly expressed in the liver were enriched in 440 and 675 GO terms in the A and B subgenomes, respectively. There were 294 GO terms shared by both gene sets. The proportions of GO terms shared by the two subgenomes was comparable for homoeologous and singleton genes (41.79% vs. 35.81%, χ^2^ test, *p* = 0.10). The DEGs in the muscle showed similar trends, in that the A and B subgenomes shared a large number of enriched GO terms (Supplementary Table S5). Furthermore, GSEA analysis revealed that the B subgenome plays a more critical role in the differentiation of tissue functions in general. Hormone biosynthesis and oxidative phosphorylation play key roles in the liver and muscle, respectively. In both pathways, genes in the B subgenome exhibited higher enrichment scores and stronger expression fold changes (Supplementary Figures S7 and S8). The lowest enrichment scores were observed in singleton genes. However, complement and coagulation cascades, which are the main components of innate immunity and adaptive immunity [20], were an exception (Figure 1F). The enrichment scores of homoeologous genes were lower than those of singletons, and both subgenomes contributed equally.

### 2.2. Asymmetric DNA Methylation Did Not Contribute to Biased Expression of Homoeologous Genes

A total of 598 million BS-seq reads were generated for liver and muscle tissues, 99.99% of which were clean data (Supplementary Table S6). On average, 68.12% of reads were uniquely mapped to the genome, and 73.52% of reads were aligned overall. The average depths of BS-seq Reads were 11.92× and 12.04× for liver and muscle tissue, respectively. According to the sequence reads mapped to mitochondrial sequences, bisulfite conversion efficiency was greater than 99.70% for each sample. According to these results, the quality of the sequencing data was sufficient to support downstream analysis.

The distribution pattern was quite similar for all three sequence contexts (CG/CHG/CHH, H representing A, C, or T), with all being more prevalent at chromosomal ends (Supplementary Figure S9). However, DNA methylation levels in CpG sites were substantially greater than those in CHH and CHG sites in liver and muscle tissue. The distributions of DNA methylation in muscle and liver tissue were also consistent, and no large-scale variations were found. 

**Figure 2:**
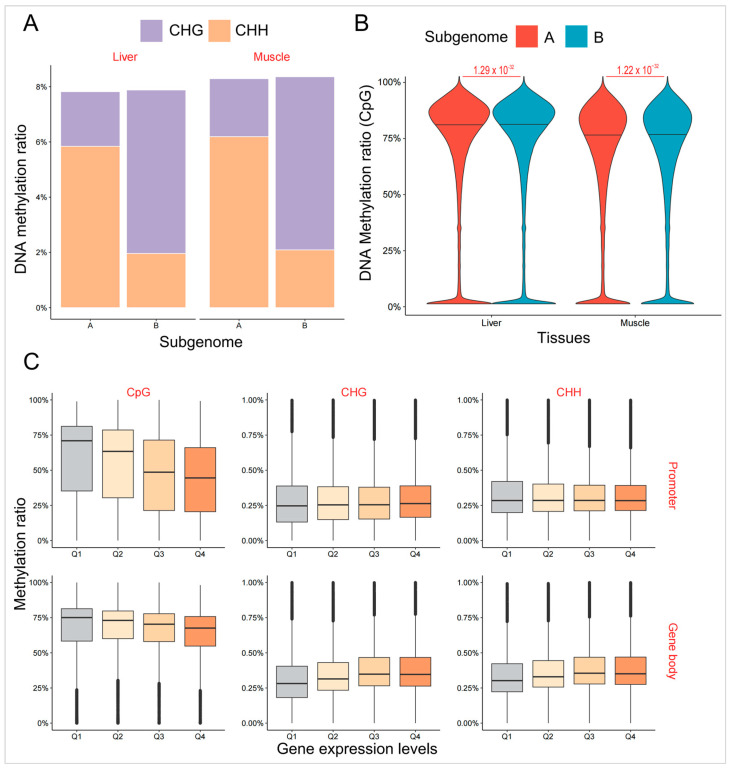
Asymmetric DNA methylation in the A and B subgenomes. (**A**) The proportion of methylated cytosines with different sequence contexts in the two subgenomes. (**B**) Comparison of DNA methylation levels of cytosines within CpG sequence context between the two subgenomes in the common carp. (**C**) DNA methylation levels of genes with different expression levels in the promoter and gene body. Gray represents genes that are not expressed, while the shade of orange represents gene expression levels.

The two subgenomes showed evidence of asymmetric DNA methylation modifications. The proportions of CHG and CHH in all methylation sites differed significantly between the two subgenomes (Figure 2A). The proportion of methylated CHG in the A subgenome (1.98% in the liver and 2.10% in the muscle) was significantly lower than that in the B subgenome (5.92% in the liver and 6.27% in the muscle, χ^2^ test, *p* < 2.20 × 10^−16^). In contrast, CHH showed the opposite trend (5.84% vs. 1.96% in the liver, 6.19% vs. 2.09 in the muscle, χ^2^ test, *p* < 2.20 × 10^−16^). No significant difference was found in the proportion of methylated CpG sites (χ^2^ test, *p* = 0.76). Moreover, the whole DNA methylation level of the A subgenome was significantly lower than that of the B subgenome, regardless of the sequence context (Figure 2B, Supplementary Figure S10, Wilcoxon rank-sum test, *p* < 2.20 × 10^−16^). DNA methylation levels of the promoter and gene body were also compared between the two subgenomes. In the B subgenome, DNA methylation levels in the promoter region were greater than those in the A subgenome (Supplementary Figures S11 and S12, Wilcoxon rank-sum test, *p* < 0.01). In contrast, genes originating from the A subgenome had higher levels of DNA methylation of CHG and CHH in the gene body (Wilcoxon rank-sum test, *p* < 10^−10^). DNA methylation levels of CG contexts in the gene body did not exhibit a significant difference between the two subgenomes (Wilcoxon rank-sum test, *p* > 0.05).

To investigate the role of DNA methylation in gene expression in the common carp, we quantified the DNA methylation levels of genes with different expression levels (Figure 2C). In the promoter, DNA methylation levels of CpG sites were significantly negatively correlated with gene expression levels (Spearman’s rank correlation test, r = −0.21 in the liver and −0.22 in the muscle, *p* < 2.20 × 10^−16^). The DNA methylation levels of non-expressed genes were the highest, and genes with the highest expression levels had the lowest DNA methylation. However, there was no correlation between gene expression and DNA modification levels of CHG and CHH sites in the promoter (Spearman’s rank correlation test, *p* > 0.05). In the gene body, increased DNA methylation levels of CpG sites were accompanied by a decrease in gene expression, whereas DNA modification levels of CHG and CHH were positively correlated with gene expression levels (Spearman’s rank correlation test, r = 0.14 for CHG in the liver, 0.16 for CHG in the muscle, 0.11 for CHH in the liver, and 0.16 for CHH in the muscle, *p* < 2.20 × 10^−16^).

**Figure 3:**
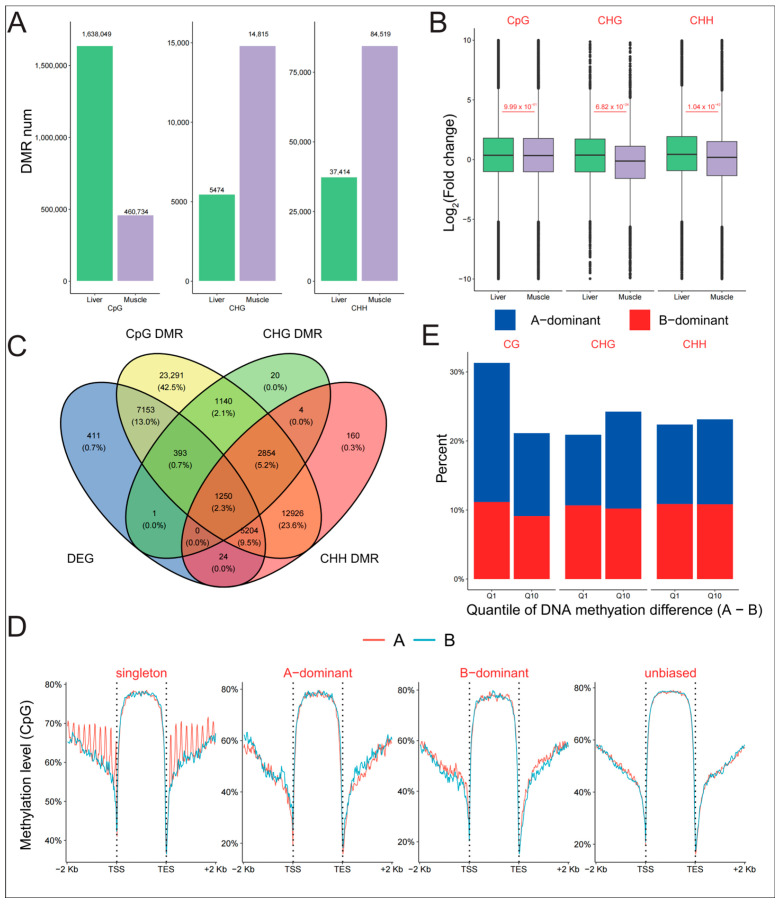
Change in DNA methylation was not correlated with homoeologous gene expression. (**A**) The number of hyper-DMRs with different sequence contexts in liver and muscle tissues. (**B**) Comparison of the fold change in expression levels of genes with different DMRs between liver and muscle tissues. (**C**) Venn diagram depicting the intersection of DEGs and genes with different DMRs. (**D**) DNA methylation levels of genes with different expression patterns. (**E**) Proportion of genes with A-/B-dominant expression in homoeologous pairs with extreme DNA methylation differences.

In the liver and muscle, the number of CpG-related differentially methylated regions (DMRs) was the highest, at 2,098,783 (Figure 3A). There were also 20,289 CHG- and 121,933 CHH-related DMRs, respectively. Approximately 78.05% of CpG DMRs were hyper-methylated in the liver, and 26.98% CHH and 30.68% CHG-related DMRs were hyper-methylated in the muscle. However, the distribution of DMRs was highly consistent across different genomic regions (Supplementary Figure S13). There were more than two-thirds of DMRs located in the gene body and promoter regions, while fewer than 25% of DMRs were found in intergenic regions, which make up the majority of the genome. To further validate the regulation of gene expression by DNA methylation, we examined the relationship between DMRs and fold changes in gene expression. The presence of CpG DMRs was not associated with fold changes in gene expression (Figure 3B, Wilcoxon rank-sum test, *p* = 0.99). However, the fold change in gene expression with hyper-CHG or CHH methylation in liver tissue was significantly greater than that of genes with hyper-DMRs in muscle tissue (Wilcoxon rank-sum test, *p* < 2.20 × 10^−16^). In addition, there were 7153 DEGs among the genes specifically harboring CpG DMRs, and 1250 DEGs among the genes with all three types of DMRs. A total of 5204 DEGs were identified among the genes with simultaneous CpG and CHH DMRs (Figure 3C), which was much higher than the number of genes with both CpG and CHH DMRs (393).

We compared the DNA methylation levels of genes with different expression patterns to examine the role of methylation in subgenome expression dominance. Singleton genes in the A subgenome had higher CpG methylation levels in the promoter (Figure 3D, Wilcoxon rank-sum test, *p* = 6.71 × 10^−5^). Genes with B-dominant expression had lower DNA methylation levels in the promoter (Wilcoxon rank-sum test, *p* < 9.98 × 10^−3^). However, there was no difference in DNA methylation level between genes with A-dominant and unbiased expression (Wilcoxon rank-sum test, *p* > 0.05). The gene body and promoter of genes with B-dominant expression were found to have hyper-CHG methylation and hypo-CHH methylation levels, respectively (Supplementary Figure S14, Wilcoxon rank-sum test, *p* < 0.001). By analyzing the relationship between methylation and expression differences between homoeologous genes, we found that only CpG methylation had a weak negative correlation with expression bias (Supplementary Figure S15, Spearman’s rank correlation test, r = −0.05 for the promoter in the liver, −0.06 for the gene body in the liver, −0.03 for the promoter in the muscle, and −0.06 for the gene body in the muscle, *p* < 0.0001). Furthermore, there was no difference in the percentage of genes with A- or B-dominant expression between the two groups with extreme methylation levels (Figure 3E, χ^2^ test, *p* = 0.08 for CG, 0.10 for CHG, 0.93 for CHH). Even in homoeologous pairs with extreme gene expression differences, there was also no significant difference in DNA methylation (Supplementary Figure S16, Spearman’s rank correlation test, *p* > 0.22). According to these findings, DNA methylation may not be involved in the regulation of subgenome expression dominance.

### 2.3. Chromatin Accessibility Was Correlated with Subgenome Expression Dominance 

Through ATAC-seq, we generated a total of 159 million reads. After filtering, 105.80 million reads were used for subsequent analysis. On average, 80.89% of the reads were mapped to the genome (Supplementary Table S7). Most insert fragments were less than 200 bp in length and exhibited typical features of DNA pitch (Supplementary Figure S17). These findings suggested that the data characterize the openness of chromatin well.

**Figure 4:**
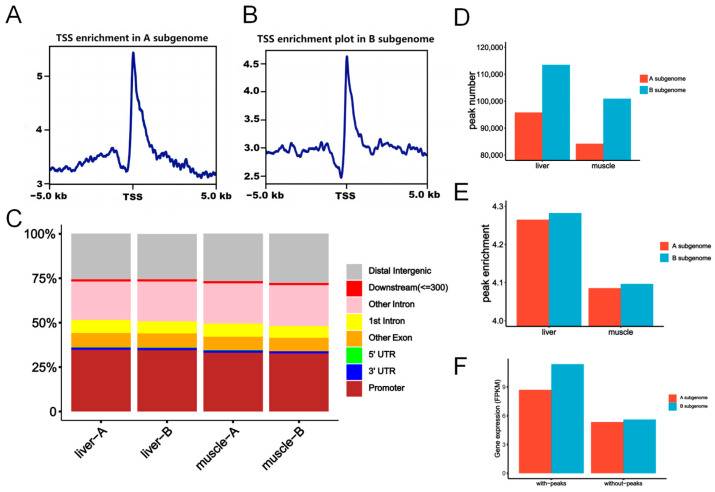
The contributions of DMRs to biased subgenome expression. ATAC-seq read enrichment plot of TSSs in the A subgenome (**A**) and the B subgenome (**B**). (**C**) Localization of ATAC peaks in different genomic regions. (**D**) The number of identified ATAC peaks in the A and B subgenomes. (**E**) The enrichment scores for peaks in the A and B subgenomes. (**F**) Gene expression with or without ATAC-seq peaks in the liver.

The distribution of chromatin accessibility on chromosomes showed that there was no significant difference between the A and B subgenomes (Supplementary Figure S18), and both subgenomes enriched ATAC-seq reads in the middle of the chromosomes. In addition, the enrichment of reads at transcription start sites (TSSs) was consistent with the typical features of ATAC-seq data (Figure 4A,B, Supplementary Figure S19). 

We identified ATAC-seq peaks and annotated them in genomic regions based on reference transcriptome annotation. The genome was divided into the promoter, CDS, 5′-UTR, 3′-UTR, intron, downstream, and distal intergenic regions. More than 30% of ATAC-seq peaks were in the promoter region, which suggested their potential function in gene expression regulation. The distribution of ATAC peaks was highly similar between liver and muscle tissue, as well as between the A and B subgenomes (Figure 4C).

We examined the number and enrichment of ATAC-seq peaks in the two subgenomes. There were more ATAC peaks in the B subgenome than in the A subgenome (Figure 4D). In the liver, 95,762 and 113,444 ATAC peaks were identified in the A and B subgenomes (χ^2^ test, *p* < 2.20 × 10^−16^), respectively. In muscle tissue, the number of peaks in the B subgenome (100,990) also was higher than that of the A subgenome (84,193, χ^2^ test, *p* < 2.20 × 10^−16^). Similarly, the enrichment of ATAC peaks was also higher in the B subgenome (Figure 4E). The analysis of peak abundances demonstrated that in the liver, the average peak enrichment for the B subgenome (4.282) was significantly higher than that for the A subgenome (4.265, Wilcoxon rank-sum test, *p* = 6.16 × 10^−8^). Similar trends were observed in muscle tissue (Average abundances: 4.085 for the A subgenome and 4.096 for the B subgenome, respectively; Wilcoxon rank-sum test, *p* = 9.02 × 10^−5^). These findings collectively indicate an increase in chromatin accessibility in the B subgenome compared with the A subgenome.

To study the relationship between chromatin accessibility and gene expression levels, we classified total genes according to the presence of ATAC peaks in the gene body or promoter. The results showed that the expression levels of genes with ATAC peaks were significantly greater than those of genes without peaks in the liver and muscle (Figure 4F; Supplementary Figure S20, Wilcoxon rank-sum test, *p* < 2.20 × 10^−16^). Moreover, the expression levels of genes associated with peaks in the B subgenome were significantly higher than those in the A subgenome in both tissues (Wilcoxon rank-sum test, *p* < 2.20 × 10^−16^). Surprisingly, there was no significant difference in the expression of genes without peaks between the A and B subgenomes in the two tissues (Wilcoxon rank-sum test, *p* = 0.48).

## 3. Discussion

Due to it being an allotetraploid, previous studies have highlighted the presence of subgenome expression dominance in the common carp [16,17]. However, the functions of homoeologous genes exhibiting biased expression patterns have remained unexplored. The conventional understanding is that these genes are associated with divergent functions, ultimately resulting in subgenomes with distinct roles. Observations in goldfish [21] and *Cyprinidae* [22] indicated that different subgenomes were associated with different traits. In contrast, our study revealed an expression pattern characterized by dominant expression of the B subgenome but common expression changes in different tissues. The distinctive expression pattern of homoeologous genes reiterates the lack of substantial differentiation in gene function in the common carp [16]. Further, functional analyses of DEGs revealed a collaborative rather than complementary or opposite relationship among these homoeologous genes. Compared with singleton genes that lost their counterparts, homoeologous genes exhibited higher expression levels and more biological functions. These results not only underscore the limited subgenome functional differentiation in the common carp, but also suggest the possibility of long-term stable coexistence of redundant homoeologous genes. Notably, singleton genes were equally engaged and had even stronger enrichment scores in the immune response pathway. The consistency in the gene functions of singleton and homoeologous genes suggests a potential link to extensive environmental adaptations of the common carp, shedding light on the intricate regulatory mechanisms that underlie the ability to navigate stress pathways [16,23]. These findings challenge prevailing assumptions about subgenome dominance and underline the importance of considering collaborative interactions among homoeologous genes in shaping the functional landscape of polyploids, such as the common carp.

Subgenome expression dominance usually occurs only in allotetraploids and interspecific hybrids [5]. It is generally accepted that TE contents guide the selection of subgenomes with dominant expression [5,6,24]. The intricate association between TEs and heritability endows DNA methylation with the potential to function during hybridization and polyploidization [25]. The observed correlation between DNA methylation and biased expression of homoeologous genes in polyploids has been well documented in numerous studies [3,5,6,24]. As regulator of gene expression, DNA methylation extensively participates in X chromosome silencing and gene suppression in a parent-of-origin-specific manner [26]. In light of this, it is reasonable to assume that DNA methylation may play a role in the regulation of subgenome expression dominance. Another piece of evidence is that autotetraploids with comparable transposon loads and DNA methylation levels usually exhibit balanced expression levels [27]. However, similar TE contents and different DNA methylation levels are simultaneously observed in the common carp. There is also evidence that DNA methylation differences in neotetraploids are not sufficient to initiate biased homoeolog expression [28]. This implies that a reassessment of the connection between DNA methylation and subgenome expression dominance is warranted. Our study of the common carp detected significant differences in DNA methylation between the two subgenomes, and a notable negative correlation was observed between DNA methylation and overall gene expression levels. However, changes in DNA methylation did not correlate with differences in gene expression among homoeologous genes. Further, no corresponding alterations in DNA methylation were identified in homoeologous gene pairs with switched expression patterns in different tissues. These intriguing findings challenge the conventional notion that DNA methylation is intricately linked to subgenome expression dominance [5,6]. However, several recent reports have also suggested that DNA methylation may not be responsible for subgenome expression dominance [28]. Our study addressed this assumption and suggested a re-conceptualization of the relationship between DNA methylation and subgenome expression dominance.

Chromatin accessibility is a direct characterization of the effect of chromatin structure on gene transcription. Advances in technology have afforded unprecedented accuracy in portraying chromatin accessibility [11]. There is significant variation in chromatin accessibility among subgenomes in polyploids [14]. Further, both subgenome expression levels and homoeologous expression bias show a robust correlation with chromatin accessibility in promoter regions [15,29]. However, the association between chromatin accessibility and subgenome dominant expression has not been documented in the common carp. Our study revealed a robust correlation between chromatin accessibility and gene expression, coupled with discernible differences between the two subgenomes. This correlation suggested an important role of chromatin accessibility in shaping subgenome expression dominance. The integration of chromatin accessibility data with gene expression profiles not only reaffirmed a strong correlation across subgenomes but also revealed compelling results. Genes lacking chromatin accessibility exhibited similar expression levels in both the A and B subgenomes. This finding implies the presence of a common regulatory mechanism governing gene expression, in which dosage-sensitive genes may serve as initiators of gene biased expression. In addition, the intricate interplay of cis-regulatory elements, characterized by interaction capacities, can also form a regulatory network that leads to subgenome expression differences [30]. Large-scale identification of cis-regulatory elements and studies of their function will provide a more definitive answer to the origin of subgenome expression dominance in the common carp.

## 4. Materials and Methods

### 4.1. Experimental Fish and Tissue Collection

Healthy one-year-old common carp var. ‘Songpu’ were obtained from the hatchery station of the Chinese Academy of Fishery Sciences and were anesthetized with MS222 (40 mg/L) before surgery. The muscles are the most commonly consumed parts of the common carp, while the liver plays an important role in metabolism and immunity [18]. Therefore, muscle and liver tissues were collected and rapidly frozen in liquid nitrogen. Each sample was prepared by mixing three individual tissues, and a total of three independent biological replicates were generated. All the experiments were performed in accordance with an animal protocol approved by the Animal Care and Use Committee of the Chinese Academy of Fishery Sciences (ACUC-CAFS).

### 4.2. RNA Extraction and Sequencing

Total RNA was extracted from each frozen tissue sample with TRIzol (Thermo Scientific, Waltham, MA, USA) according to the manufacturer’s instructions. Genomic DNA was removed using DNase I (BioFit Co., Chengdu, China). The RNA quality of each sample was assessed using a Nanodrop 2000 spectrophotometer (Nanodrop, Wilmington, DE, USA) with 1% agarose gel and Agilent 2100 Bioanalyzer (Agilent Technologies, Santa Clara, CA, USA). A total of 1 µg high-quality RNA per sample was used and RNA-seq libraries were prepared using the TruSeq RNA Library Prep Kit v2 (Illumina, San Diego, CA, USA) according to standard procedures. Briefly, mRNA was purified from total RNA using oligo-dT attached magnetic beads. Then, mRNA was fragmented and used as a template for cDNA synthesis. cDNA products were subjected to end-repair process, ‘A’ base addition, and adapter ligation, and then purified and enriched via PCR amplification. Finally, the prepared libraries were sequenced on HiSeq 4000 (Illumina, San Diego, CA, USA) platform to generate 150 bp paired-end reads.

### 4.3. Quantification of Gene Expression and Identification of Differentially Expressed Genes

Fastp v0.21.0 [31] was used to filter out adaptor sequences, low-quality bases, and reads shorter than 90 bp from the raw sequencing reads. Reads that passed quality control were subsequently mapped to the common carp reference genome (NCBI RefSeq accession: GCF_018340385.1) using HISAT v2.2.1 [32] with default parameters. The gene expression of each sample was estimated using Stringtie v2.2.1 [33] with reference transcriptome annotation (NCBI RefSeq accession: GCF_018340385.1) and normalized to the fragments per kilobase of transcript per million mapped read (FPKM) values. 

Reads aligned to genes were counted using featurecount v2.0.1 [34] with the parameters ‘–t exon –g gene_id’. The read count matrix was subsequently imported into DESeq2 v1.28.1 [35] for differential expression analysis based on negative binomial distribution. Genes with a logarithmic fold change absolute value greater than 2 and FDR less than 0.05 were considered DEGs. GO enrichment analyses of DEGs were carried out using TBtools v2.012 [36], and *p* values were adjusted using the Benjamini–Hochberg method. Gene set enrichment analysis was implemented by GSEA v4.2.1 [37]. Statistical analysis was conducted using R v4.2.3, and data visualization was performed using ggplot v3.4.1.

### 4.4. Whole-Genome Bisulfite Sequencing (WGBS)

Genomic DNA was extracted from frozen tissues using SS Kit and bisulfite was processed using EZ DNA Methylation-Gold Kit (Zymo Research, Orange, CA, USA). Bisulfite-converted DNA fragments were subjected to PCR amplification, purification, and library preparation following the manufacturer’s instructions. The qPCR-based method was used to quantify the constructed DNA libraries, and sequencing was performed on an Illumina NovaSeq 6000 platform in PE150 mode at Berry Genomics (Beijing, China). 

### 4.5. DNA Methylation Sequencing Data Analysis

Fastp v0.21.0 was used to filter adapter sequences and low-quality reads in paired-end reads mode. The filtered reads were aligned to the common carp reference genome using BSMAP v2.6 with the following parameters: -v 15 -r 2 -p 40 -n 0 -x 1000 [38]. SAMtools v1.4 [39] was used to determine the genome-wide coverage and average depth of DNA methylation sequencing reads. After removing PCR duplicates and reads with a quality score less than 20, DNA methylation levels and sequence contexts for each cytosine were determined using BatMeth2 v2.1 [40]. DNA methylation profiles for gene body and 2 Kb promoter regions were generated using methyGff module and visualized with bt2profile.py script. 

DMRs were identified with 600 bp sliding windows with a step size of 200 bp. DNA methylation levels of the sliding windows were measured in three different sequence contexts, including CpG, CHG, and CHH, respectively. MethylKit v1.16.1 [41] was used to calculate differential methylation, and *p* values were adjusted using the sliding linear model method. DMRs were identified with an adjusted *p* value cutoff of 0.05. Annotating the DMR regions and identifying the nearby genes was conducted using ChIPseeker v1.26.2 [42].

### 4.6. Chromatin Accessibility Profiling by ATAC-Seq 

Assay for transposase-accessible chromatin using sequencing (ATAC-seq) libraries were prepared by the hyperactive Tn5 transposase using Tn5 DNA Library Prep Kit (APExBIO, Shanghai, China) according to the manufacturer’s protocol. For each sample, approximately 200 mg of tissue was lysed to obtain the pellet nuclei. The nuclei were then incubated with transposase enzyme in tagmentation buffer at 37 °C for 30 min. The reaction products were purified using MinElute Reaction Cleanup Kit (Qiagen, Hilden, Germany) and amplified for 10 cycles using a unique combination of i7 indexed primer and i5 indexed primer, followed by purification with beads. The quality of the constructed DNA libraries was evaluated using Agilent 2100 Bioanalyzer (Agilent Technologies, Santa Clara, CA, USA) and sequencing was performed on NovaSeq 6000 (Illumina, San Diego, CA, USA) in paired-end 150 bp mode.

### 4.7. Analysis of Open Chromatin Regions

Raw reads were filtered to obtain clean reads by removing adaptors, low-quality reads, and short reads using Fastp v0.21.0. Clean reads were mapped to the common carp reference genome using Bowtie2 v2.3.5 [43] with --very-sensitive -X 2000. The alignment files for each sample were sorted using SAMtools v1.4 and PCR duplicates were removed using Sambamba v0.8.1 [44]. Open chromatin region (ATAC peak) calling was performed using MACS2 v2.2.7 [45] with --shift -37 --extsize 73 -g 1.8e9. Annotation of ATAC peaks to gene features was conducted using ChIPseeker v1.26.2. Reads count, normalization, and visualization were performed using DeepTools v3.3.0 [46]. Differential ATAC peaks between two tissues were identified using DiffBind v2.16.2 [47] with a cutoff of adjusted *p* value <  0.05 and log2|fold-change| > 1.

## 5. Conclusions

To our knowledge, the common carp stands out as the first polyploidy identified to exhibit unbiased fractionation and subgenome expression dominance. In an unexpected finding, we found that gene expression patterns and functional characteristics were concordant across the two subgenomes in the common carp. Although DNA methylation is indeed involved in the regulation of gene expression, its relationship with subgenome expression dominance has yet to be further confirmed. There was a significant difference in chromatin accessibility between the two subgenomes, which may have contributed to the expression dominance of the B subgenome. The insights gained from this study not only contribute to the field of fish biology, but also offer a valuable foundation for advancing our understanding of polyploid evolution in diverse organisms.

## Data Availability

RNA-seq, BS-seq, and ATAC-seq reads of liver and muscle tissue were deposited in the NCBI SRA database (accession number: PRJNA993294).

## Supplementary Materials

The supporting information can be downloaded at: https://www.mdpi.com/article/10.3390/ijms25031635/s1.
