# Supplementary material for: DNA Methylation and Chromatin Accessibility Impact Subgenome Expression Dominance in the Common Carp (Cyprinus carpio)
Source: Int J Mol Sci. 2024 Jan 29;25(3):1635. doi: 10.3390/ijms25031635 (PMC10855618; doi:10.3390/ijms25031635)

Contents

Supplementary Figure S1. RNA-seq quality control metrics for sequencing tissues. 2

Supplementary Figure S2. The proportion of gene expressed in liver and muscle tissues. 3

Supplementary Figure S3. The expression level of genes originating from different subgenomes in liver and muscle tissues. 4

Supplementary Figure S4. Volcano plot of differentially expressed genes in liver and muscle tissues........... 5

Supplementary Figure S5. Comparison of proportion of DEGs among different subgenomes. 6

Supplementary Figure S6. Venn diagram of GO terms enriched in DEGs from different subgenomes. 7

Supplementary Figure S7. Gene set enrichment analysis of steroid hormone biosynthesis pathway. 8

Supplementary Figure S8. Gene set enrichment analysis of Oxidative phosphorylation pathway. 9

Supplementary Figure S9. Circle map of DNA methylation levels in liver and muscle. 10

Supplementary Figure S10. Comparison of DNA methylation levels between the two subgenomes in common carp. 11

Supplementary Figure S11. DNA methylation levels of gene region in liver. 12

Supplementary Figure S12. DNA methylation levels of gene region in muscle. 13

Supplementary Figure S13. Localization of DMRs in different genomic regions. 14

Supplementary Figure S14. DNA methylation levels of gene with different expression patterns. 15

Supplementary Figure S15. Correlation of gene biased expression and DNA methylation difference in homoeologous genes. 16

Supplementary Figure S16. Comparison of DNA methylation difference between genes with switched expression profiles. 17

Supplementary Figure S17. The length distribution of sequenced fragments. 18

Supplementary Figure S18. The distribution of ATAC-seq reads across total chromosomes. 19

Supplementary Figure S19. ATAC-seq reads enrichment plot of TSS in the two subgenomes. 20

Supplementary Figure S20. Comparison of gene expression with or without ATAC-seq peaks in muscle. .. 21

Supplementary Figure S1. RNA-seq quality control metrics for sequencing tissues. (**A**) PCA plot of RNA-seq data according to gene expression levels (FPKM). (**B**) RNA-seq saturation curves for the different samples. The expressed genes were defined as those with FPKM greater than 0.1.

A

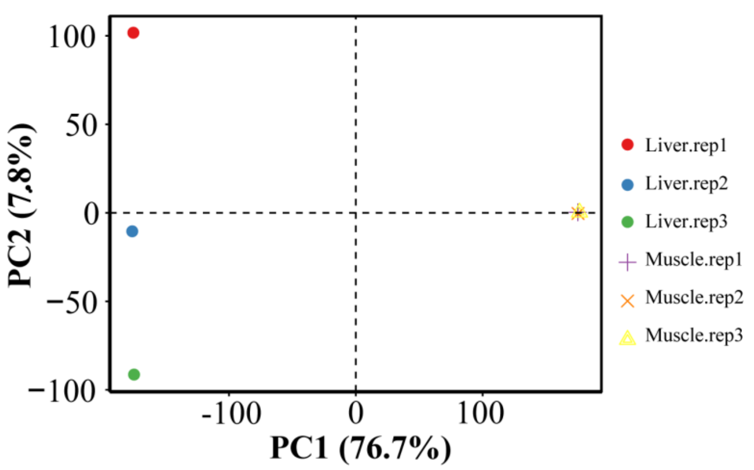

B

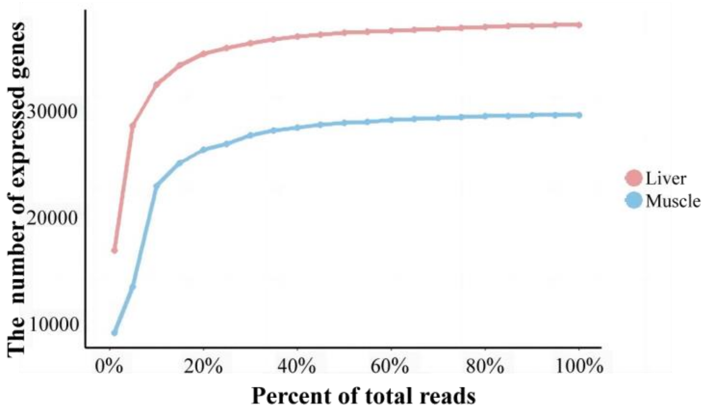

Supplementary Figure S2. The proportion of gene expressed in liver and muscle tissues.

The proportion of genes detected to expressed (FPKM > 0.1) in liver (**A**) and muscle (**B**).

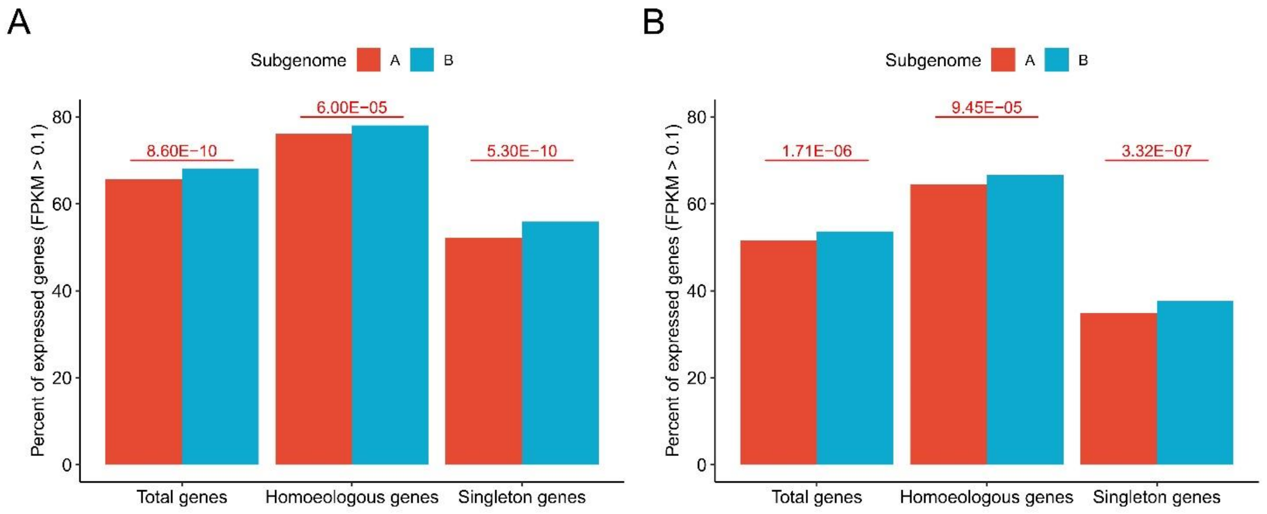

Supplementary Figure S3. The expression level of genes originating from different subgenomes in liver and muscle tissues.

The expression level (log2-transformed FPKM) of genes origating from the A and B subgenomes in liver (**A**) and muscle (**B**)

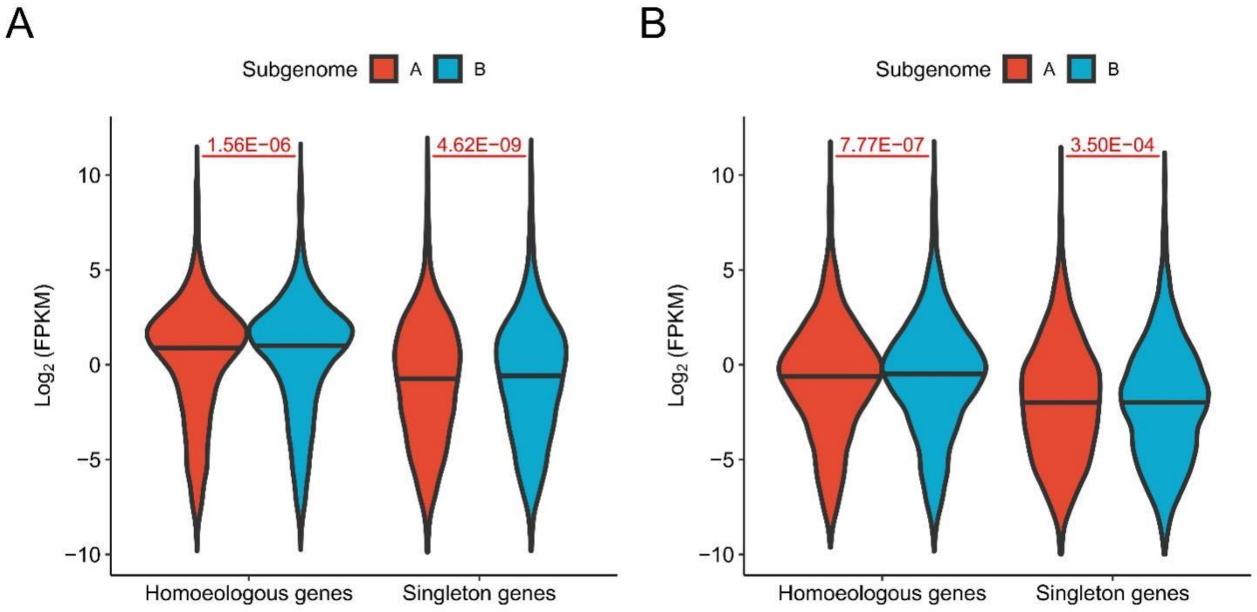

Supplementary Figure S4. Volcano plot of differentially expressed genes in liver and muscle tissues.

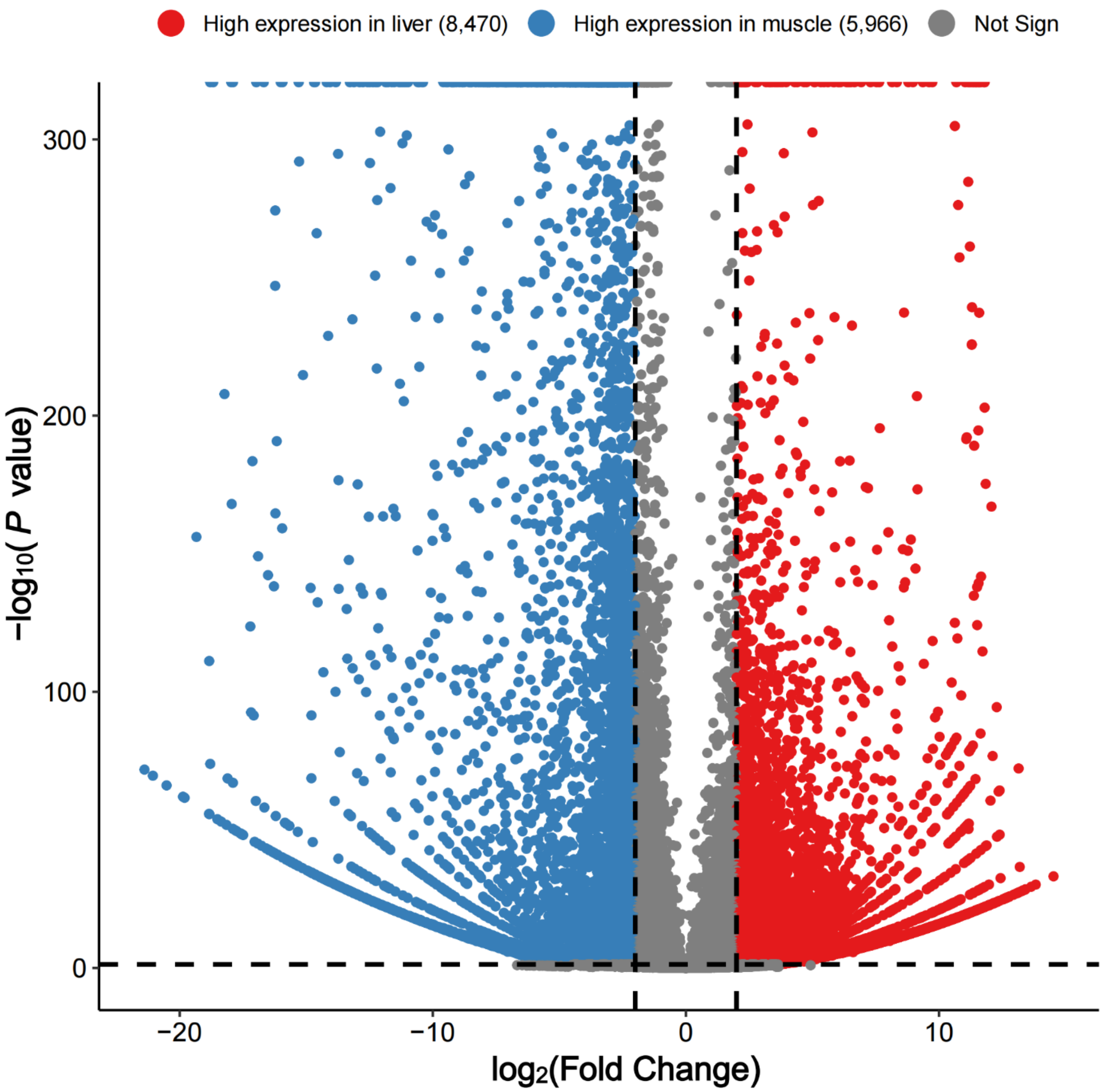

Supplementary Figure S5. Comparison of proportion of DEGs among different subgenomes.

The proportion of genes with higher expression level in liver (**A**) and muscle (**B**).

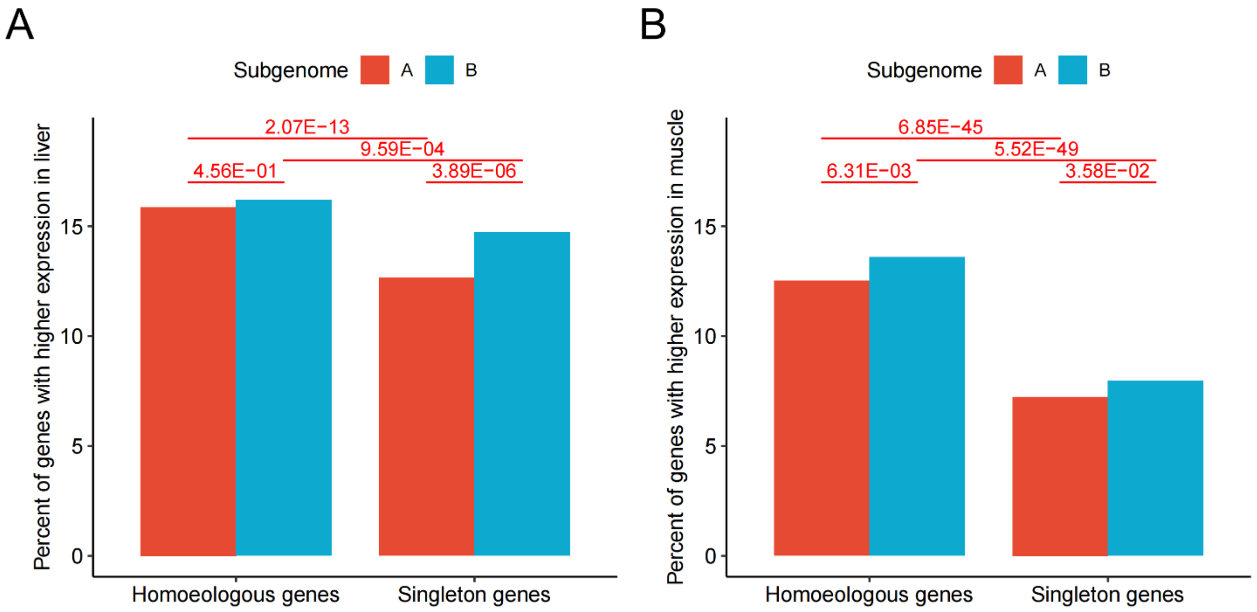

Supplementary Figure S6. Venn diagram of GO terms enriched in DEGs from different subgenomes.

The venn diagram of GO terms enriched in genes with higher expression level in liver (**A**) and muscle (**B**)

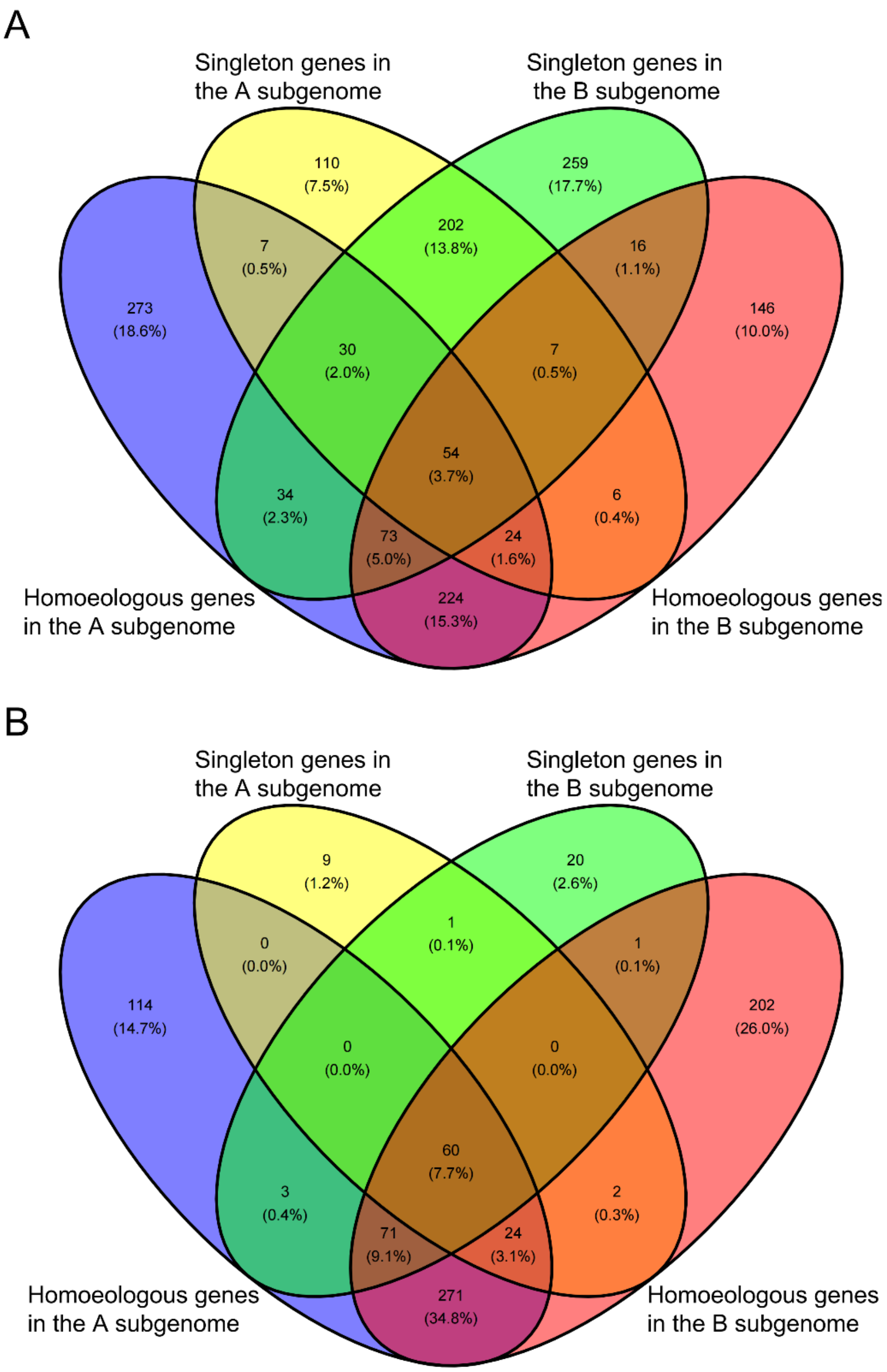

Supplementary Figure S7. Gene set enrichment analysis of steroid hormone biosynthesis pathway.

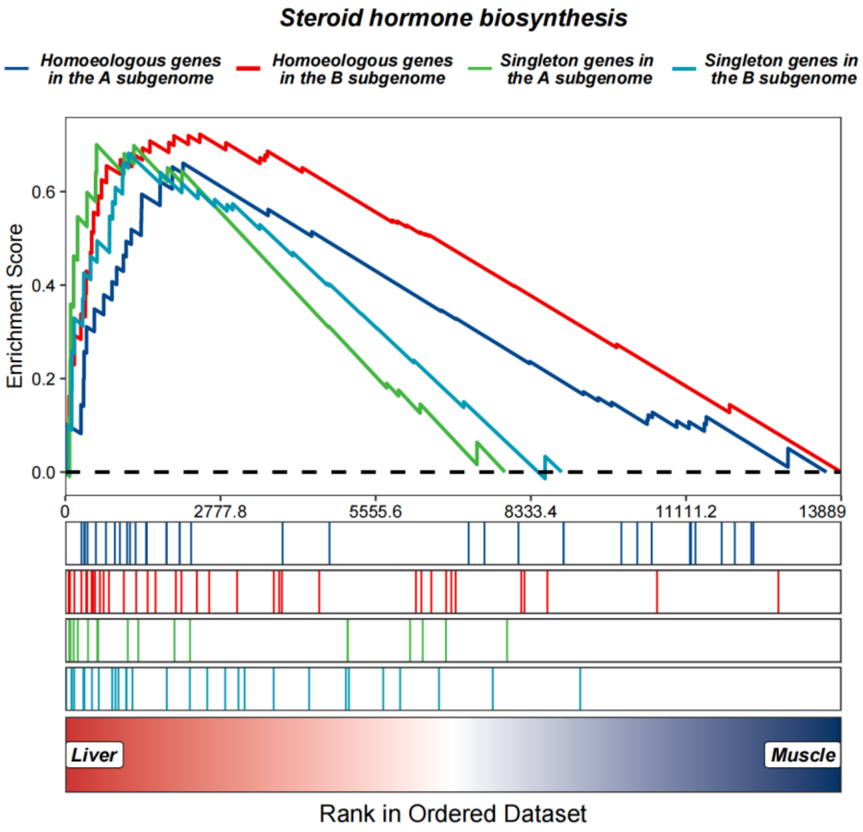

Supplementary Figure S8. Gene set enrichment analysis of Oxidative phosphorylation pathway.

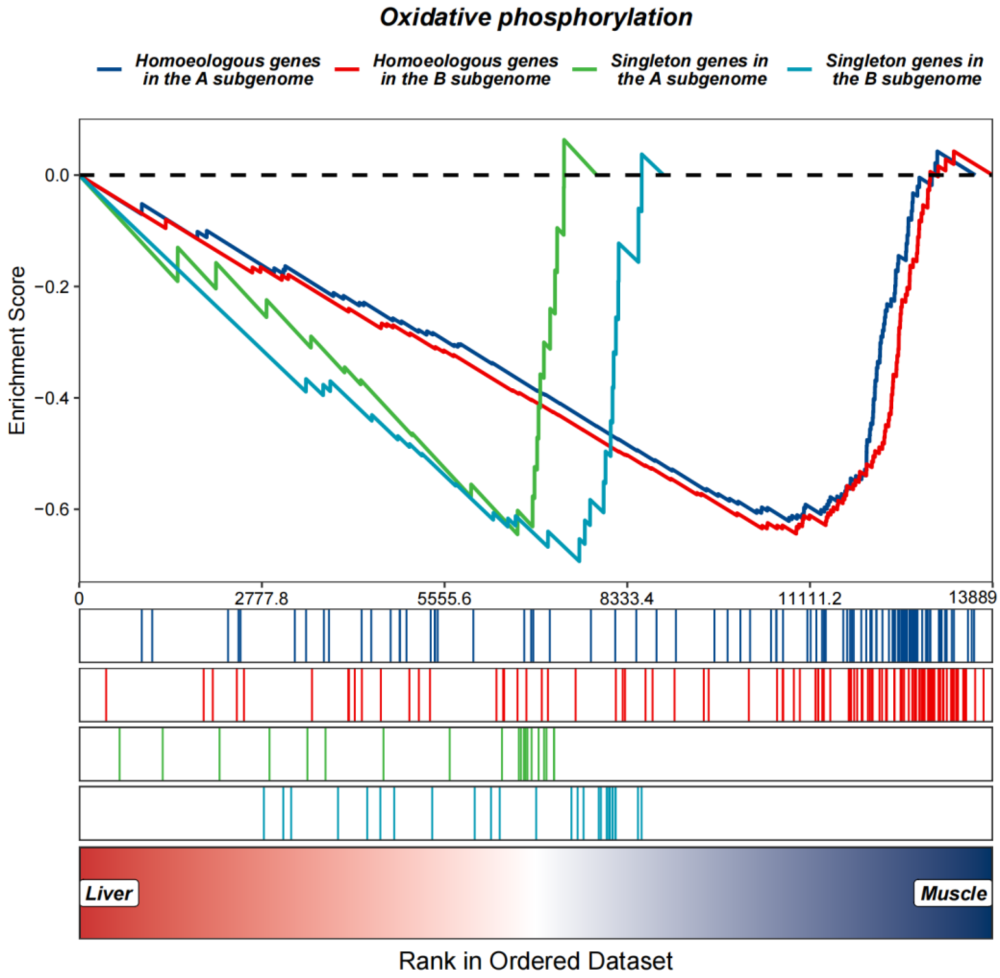

Supplementary Figure S9. Circle map of DNA methylation levels in liver and muscle.

DNA methylation levels of CpG sites in liver (A), CpG sites in muscle (B), CHG sites in liver (C), CHG sites in muscle (D), CHH sites in liver (E) and CHH sites in muscle (F) were displayed sequentially from outside to inside of the circle. DNA methylation distributions were identified using 0.1 Mb sliding windows.

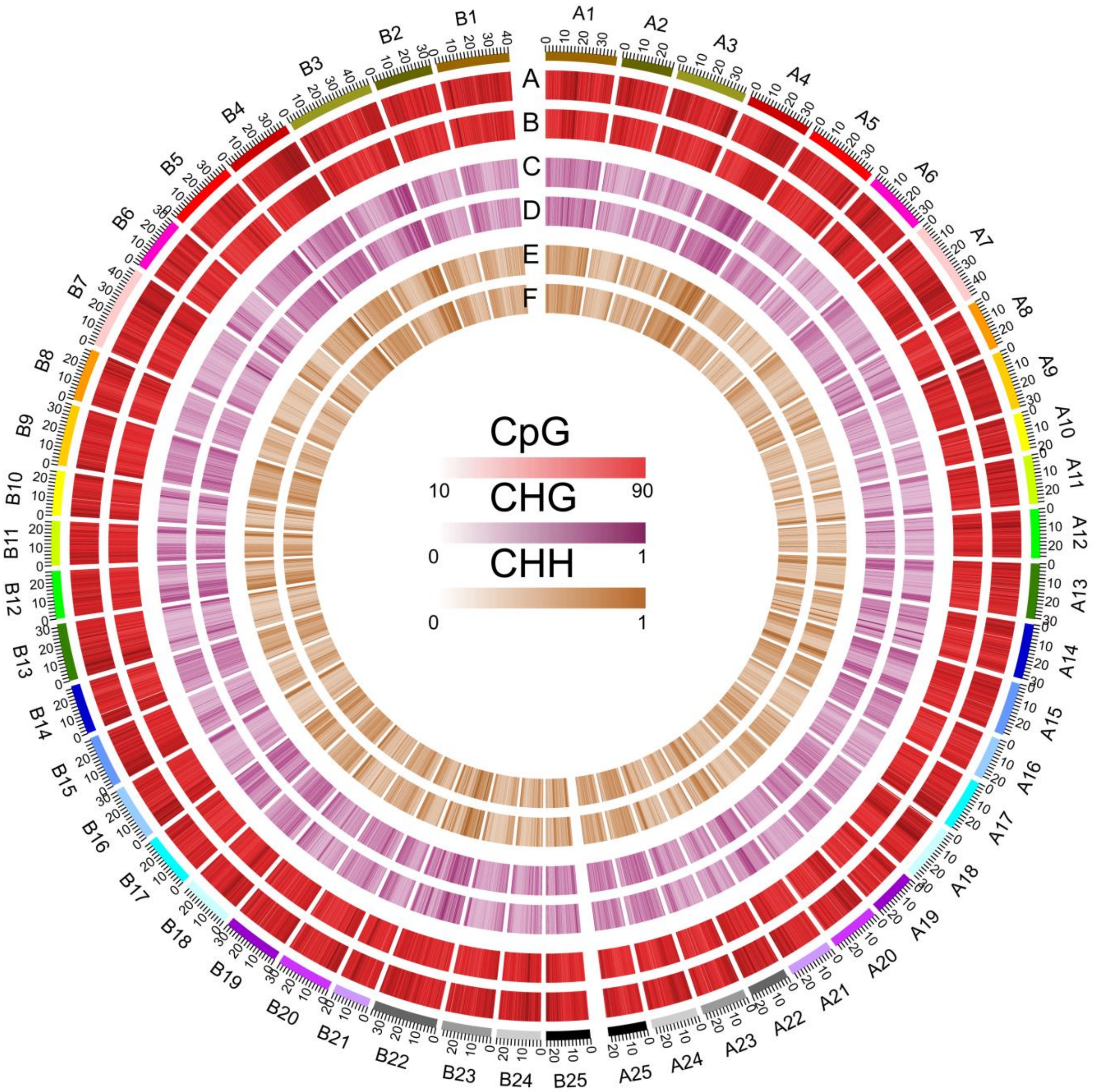

Supplementary Figure S10. Comparison of DNA methylation levels between the two subgenomes in common carp.

DNA methylation levels of CG (**A**), CHG (**B**), or CHH (**C**) in the A and B subgenomes.

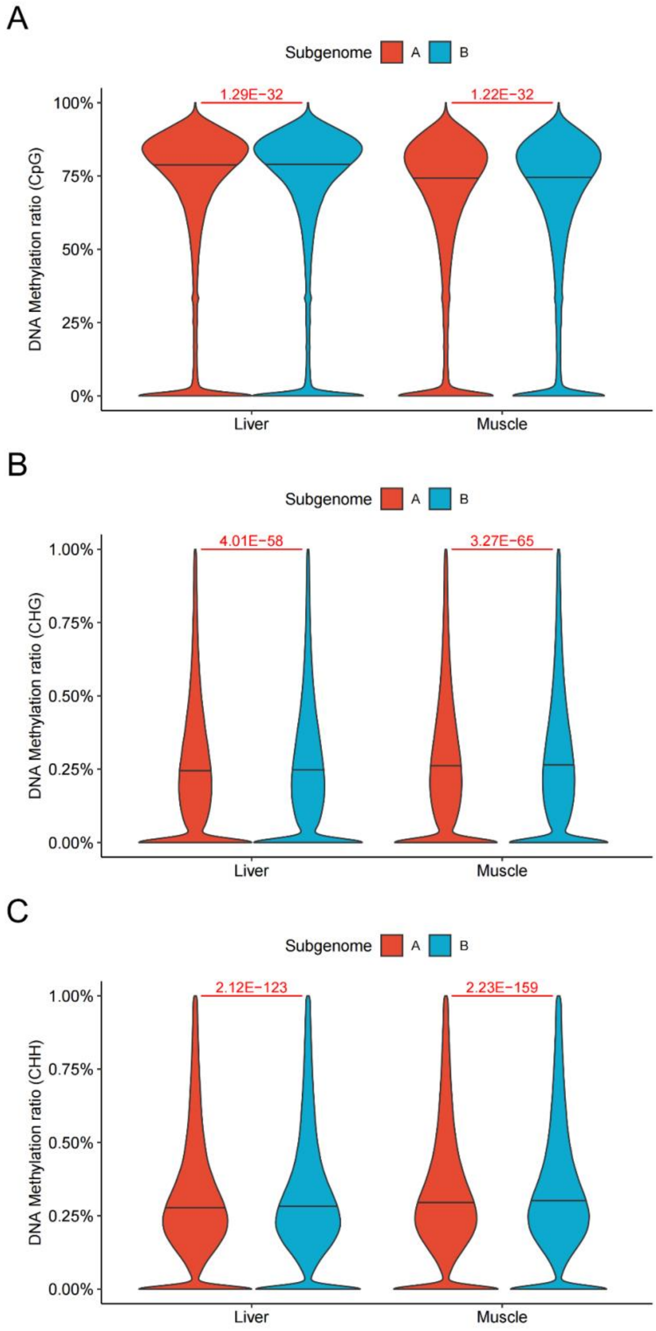

Supplementary Figure S11. DNA methylation levels of gene region in liver.

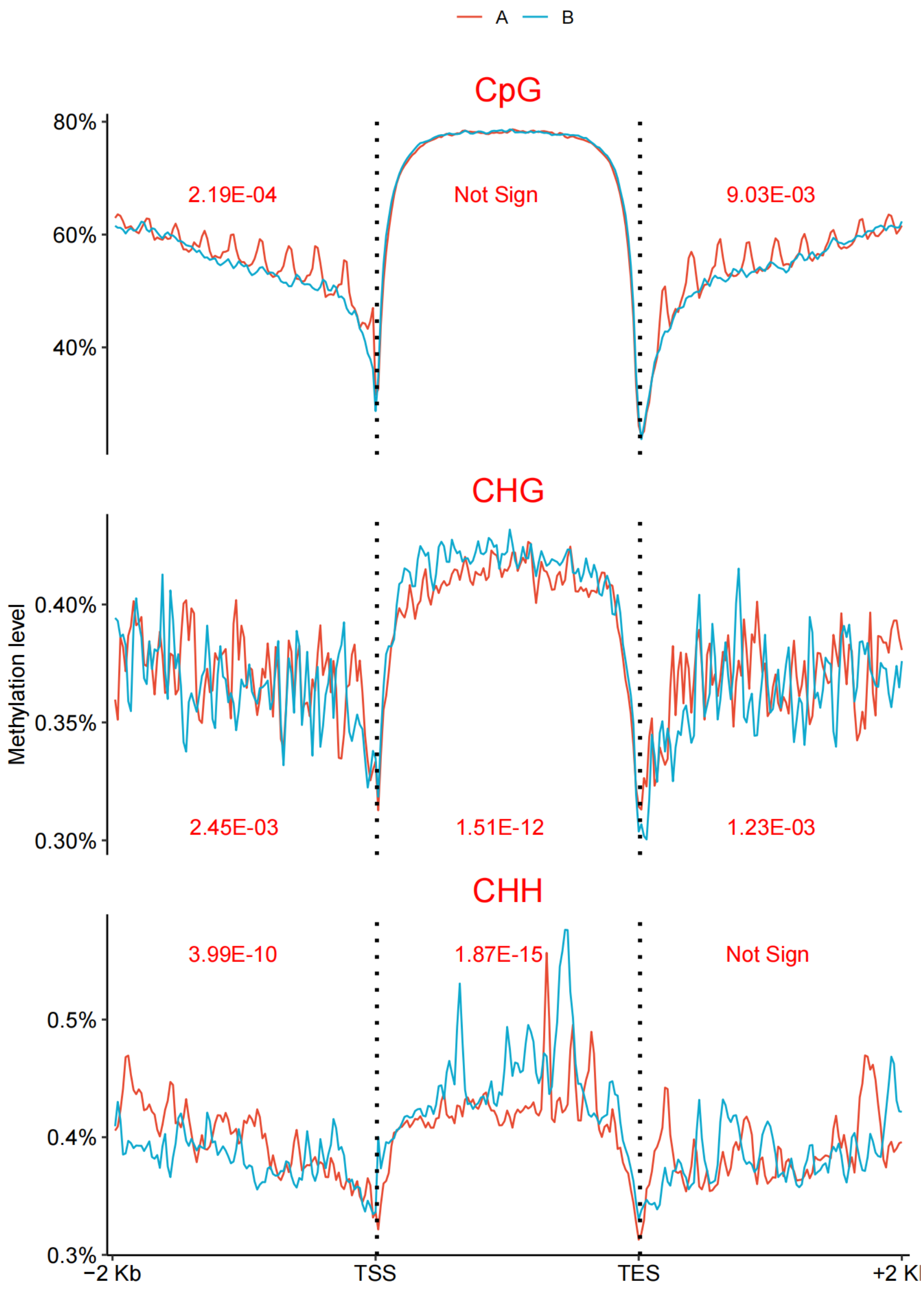

Supplementary Figure S12. DNA methylation levels of gene region in muscle.

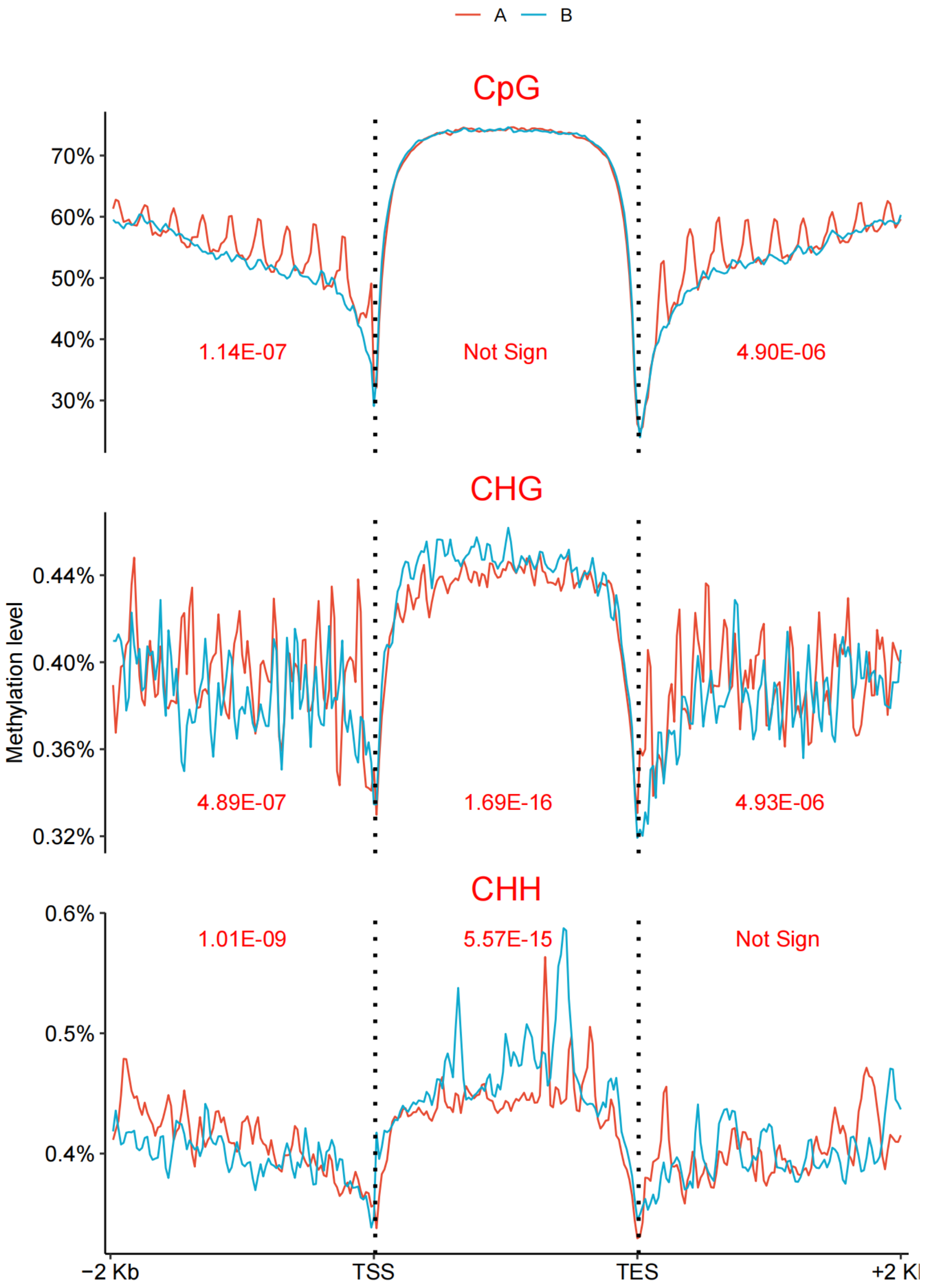

Supplementary Figure S13. Localization of DMRs in different genomic regions.

Left-panels represented the genome-wide distribution of hyper-DMRs in liver, and right-panels stand for the distribution of hyper-DMRs in muscle.

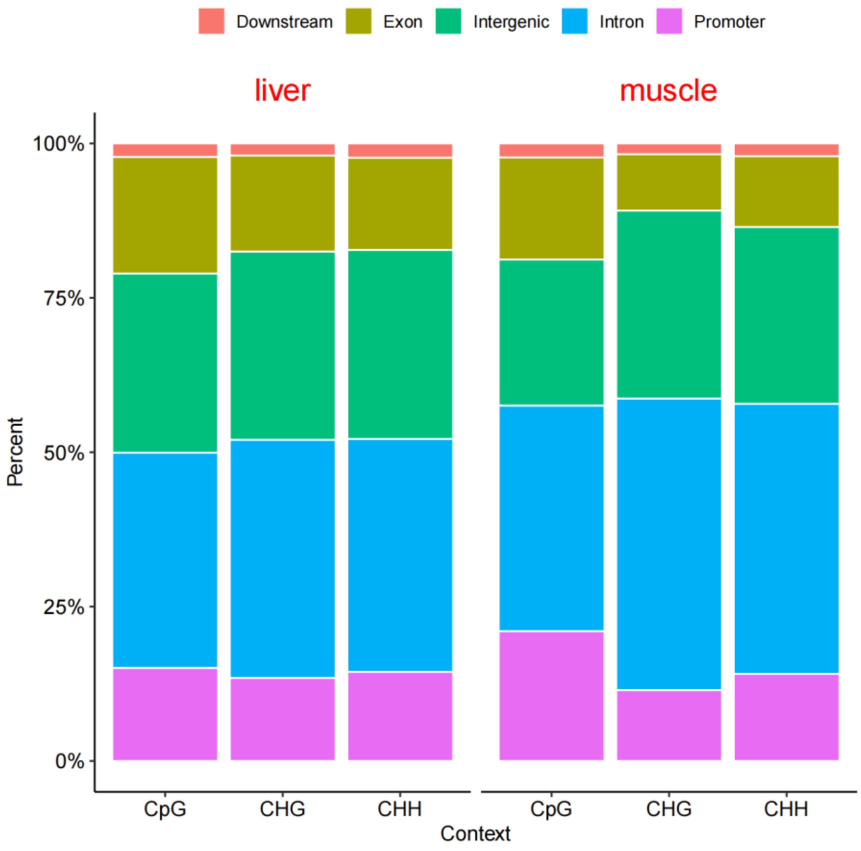

Supplementary Figure S14. DNA methylation levels of gene with different expression patterns.

DNA methylation levels of CHG (**A**) and CHH (**B**) across gene with different expression patterns.

A

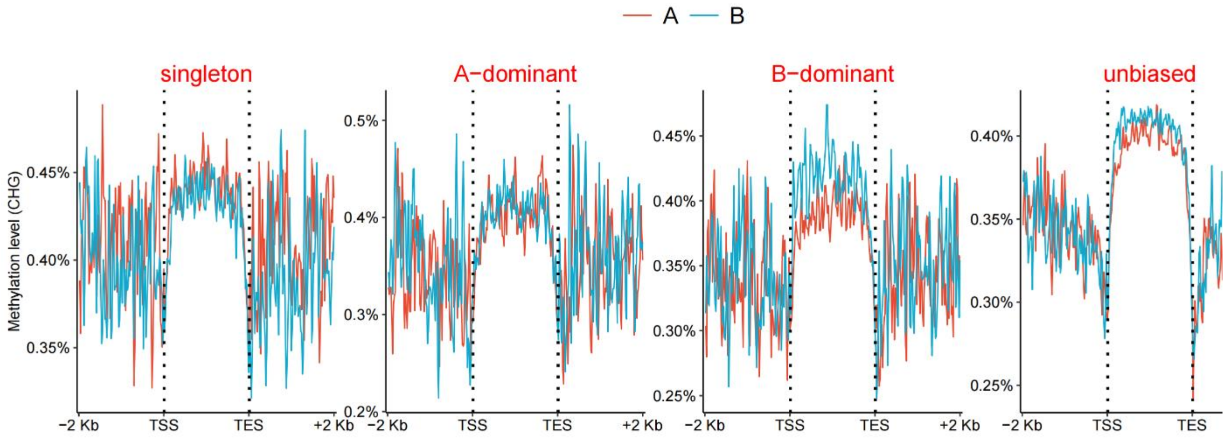

B

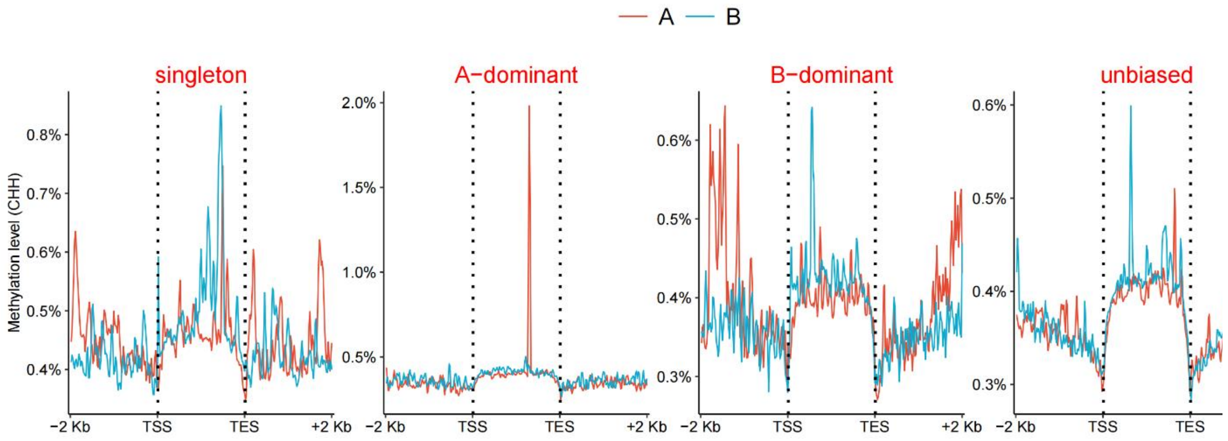

Supplementary Figure S15. Correlation of gene biased expression and DNA methylation difference in homoeologous genes.

CG methylation difference of homoeologous genes in promoter in liver (**A**), gene body in liver (**B**), promoter in muscle (**C**), gene body in muscle (**D**) was weak correlated with gene biased expression.

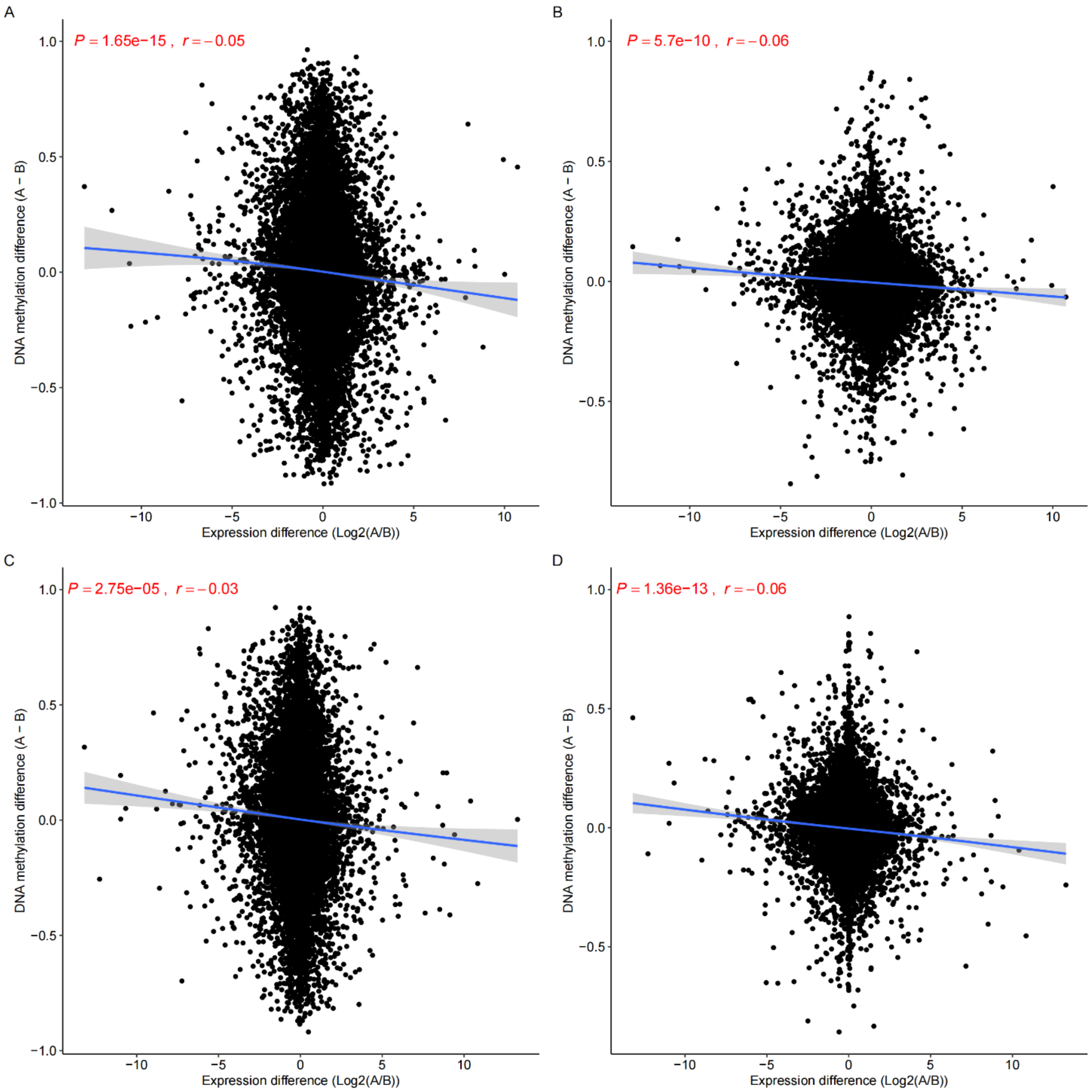

Supplementary Figure S16. Comparison of DNA methylation difference between genes with switched expression profiles.

The difference in DNA methylation levels of CG (**A**), CHG (**B**), and CHH (**C**) between different homoeologous gene pairs.

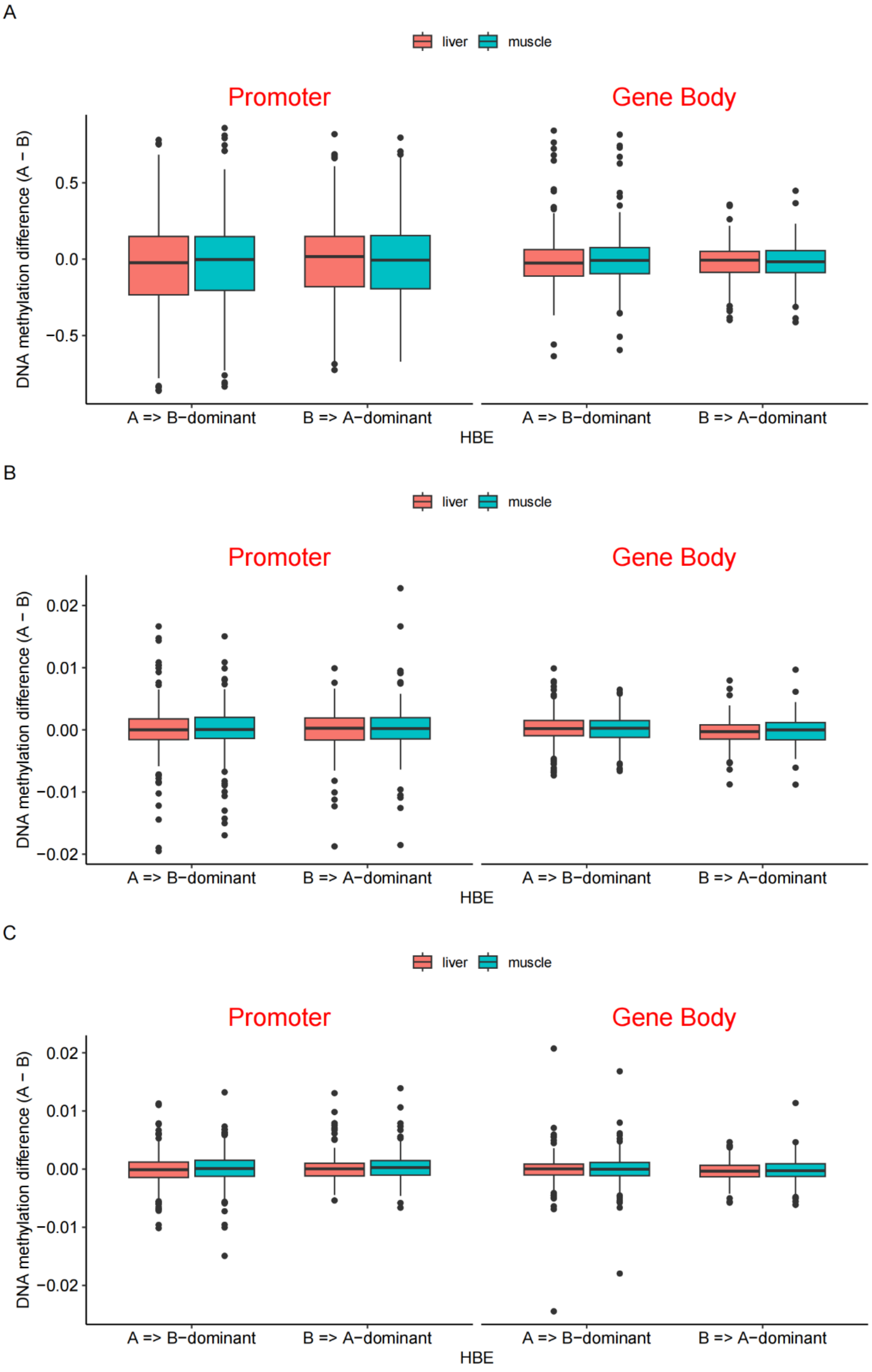

Supplementary Figure S17. The length distribution of sequenced fragments.

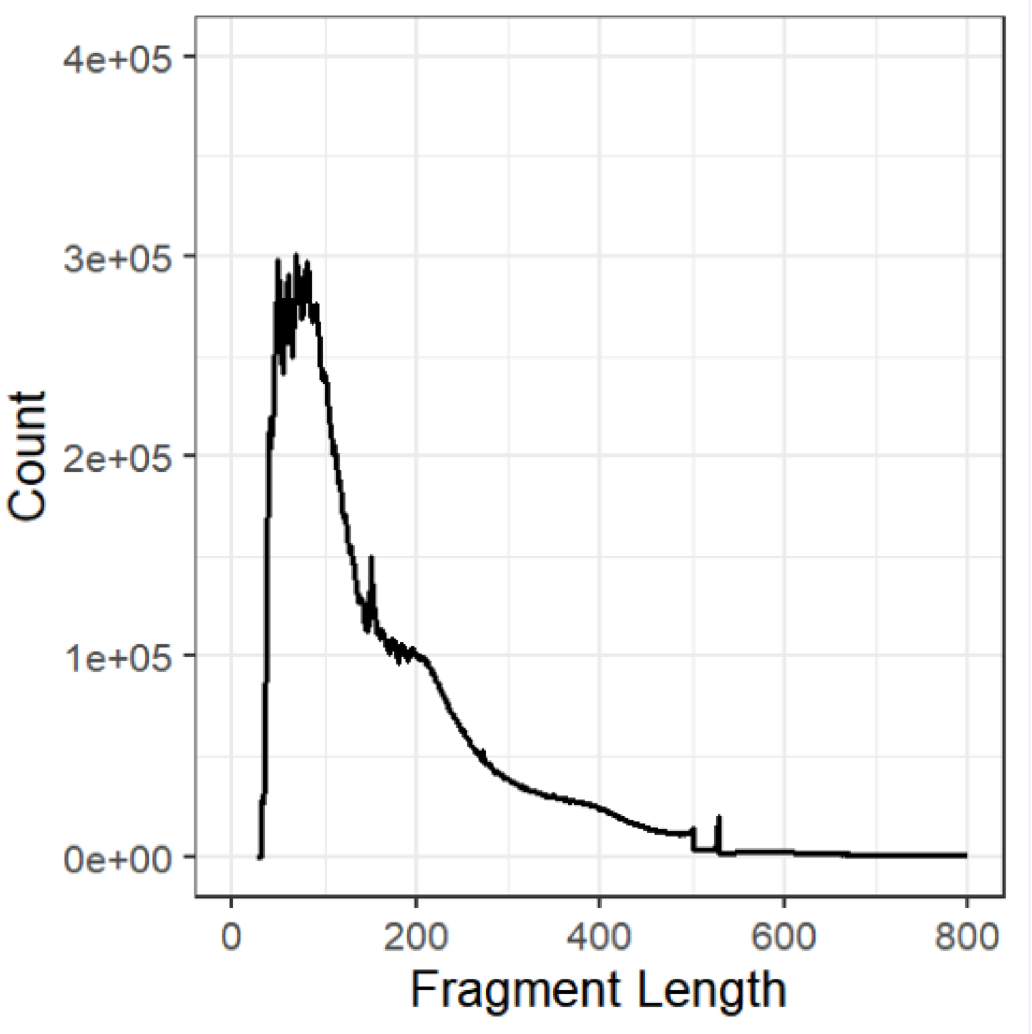

Supplementary Figure S18. The distribution of ATAC-seq reads across total chromosomes.

The genome-wide distribution of ATAC-seq reads across the A subgenome (**A**) and the B subgenome (**B**).

A

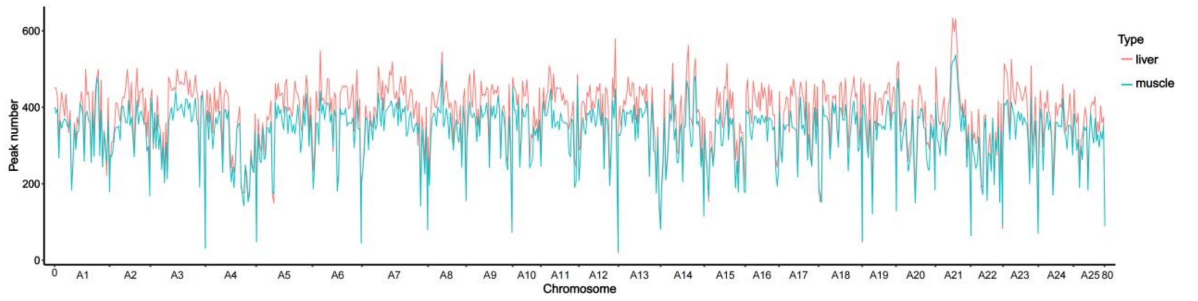

B

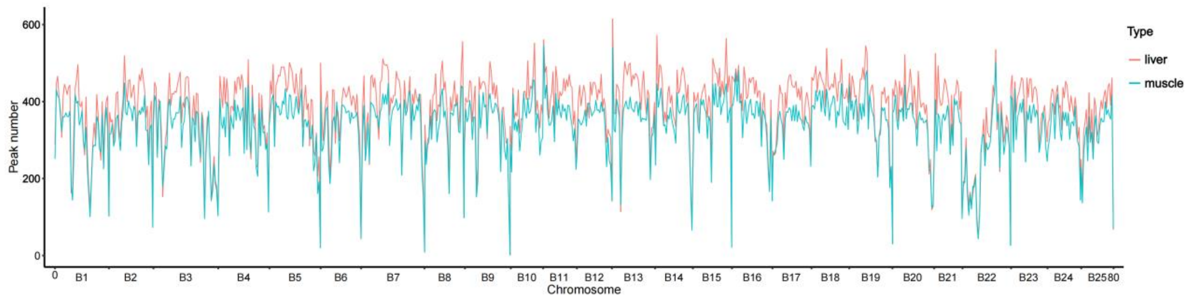

Supplementary Figure S19. ATAC-seq reads enrichment plot of TSS in the two subgenomes.

Enrichment plot of ATAC-seq reads across genes in the A (**A**) and B (**B**) subgenomes.

A	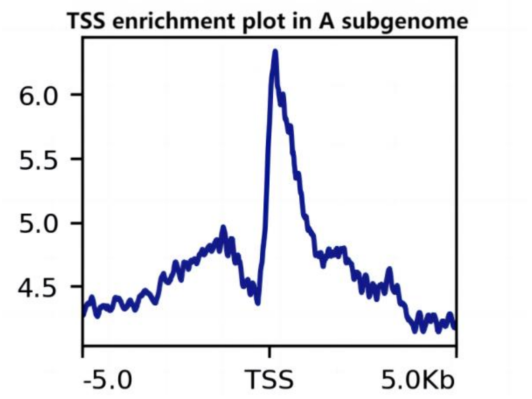
B	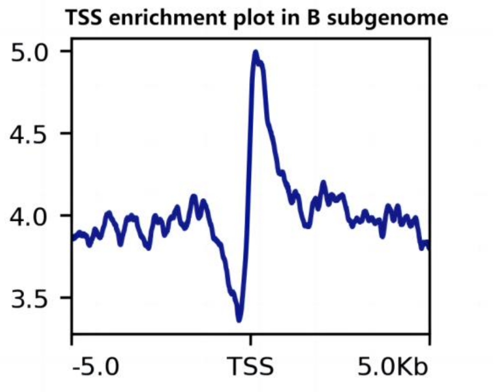

Supplementary Figure S20. Comparison of gene expression with or without ATAC-seq peaks in muscle.

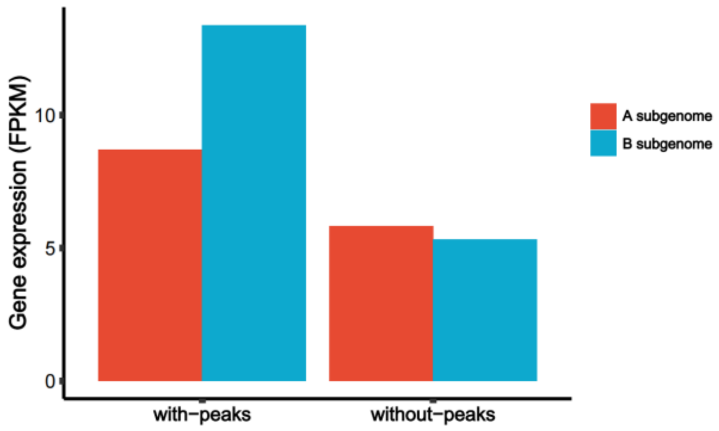

Supplement: Supplementary file 1 [file ijms-25-01635-s001.zip › Supplementary Figures.pdf]
