# Supplementary material for: DNA Methylation and Chromatin Accessibility Impact Subgenome Expression Dominance in the Common Carp (Cyprinus carpio)
Source: Int J Mol Sci. 2024 Jan 29;25(3):1635. doi: 10.3390/ijms25031635 (PMC10855618; doi:10.3390/ijms25031635)
Supplement: Supplementary file 1 [file ijms-25-01635-s001.zip › Supplementary Tables.pdf]

Contents

Supplementary Table S1. Summary of RNA-seq reads used in this study... 2

Supplementary Table S2. GO enrichment analysis of genes with significantly higher expression levels in liver than in muscle. 3

Supplementary Table S3. GO enrichment analysis of genes with significantly higher expression levels in muscle than in liver. 4

Supplementary Table S4. GO enrichment scores for different classes of genes highly expressed in liver....... 5

Supplementary Table S5. GO enrichment scores for different classes of genes highly expressed in muscle... 6

Supplementary Table S6. Summary of whole genome bisulfite sequencing statistics.................................... 7

Supplementary Table S7. Summary of ATAC-seq reads.. 8

Supplementary Table S1. Summary of RNA-seq reads used in this study.

	Liver			Muscle		
	**Replicate 1**	**Replicate 2**	**Replicate 3**	**Replicate 1**	**Replicate 2**	**Replicate 3**
Raw Reads Pairs	22,039,085	25,125,441	22,047,130	25,371,639	19,624,616	16,976,819
Clean Reads Pairs	22,013,867	25,095,090	22,021,047	25,332,516	19,593,014	16,947,641
Adapter rate (%)	1.23	1.08	0.95	0.83	0.91	0.80
Clean data rate (%)	99.89	99.88	99.88	99.85	99.84	99.82
Unique alignment rate (%)	80.68	80.69	80.71	84.82	84.85	79.20
Multiple alignment rate (%)	5.36	5.37	5.36	5.64	5.65	11.24
Chimera alignment rate (%)	7.85	7.81	7.85	6.25	6.17	3.32
Overall alignment rate (%)	93.89	93.87	93.93	96.71	96.68	93.75

Supplementary Table S2. GO enrichment analysis of genes with significantly higher expression levels in liver than in muscle.

(in a separate excel file)

Supplementary Table S3. GO enrichment analysis of genes with significantly higher expression levels in muscle than in liver.

(in a separate excel file)

Supplementary Table S4. GO enrichment scores for different classes of genes highly expressed in liver.

(in a separate excel file)

Supplementary Table S5. GO enrichment scores for different classes of genes highly expressed in muscle.

(in a separate excel file)

Supplementary Table S6. Summary of whole genome bisulfite sequencing statistics.

	Liver			Muscle		
	**Replicate 1**	**Replicate 2**	**Replicate 3**	**Replicate 1**	**Replicate 2**	**Replicate 3**
Raw Reads Pairs	108,468,166	98,138,497	89,071,557	89,086,432	100,847,226	113,060,725
Clean Reads Pairs	108,463,926	98,134,591	89,068,767	89,085,070	100,845,873	113,059,223
Clean Data Rate (%)	99.99	99.99	99.99	99.99	99.99	99.99
Mapped Pairs Rate (%)	73.90	74.00	74.10	72.90	73.00	73.20
Unique Mapped Pairs Rate (%)	68.60	68.60	68.70	67.50	67.60	67.70
Non-Unique Mapped Rate (%)	5.30	5.30	5.40	5.40	5.40	5.50
Conversion Efficiency (%)	99.73	99.72	99.71	99.70	99.72	99.71

Supplementary Table S7. Summary of ATAC-seq reads.

	Liver			Muscle		
	**Replicate 1**	**Replicate 2**	**Replicate 3**	**Replicate 1**	**Replicate 2**	**Replicate 3**
Raw Reads Pairs	25,747,154	27,677,904	27,530,236	29,446,998	26,407,728	23,093,350
Clean Reads Pairs	16,256,034	18,300,094	16,240,925	20,183,932	19,299,924	15,516,015
Clean data rate (%)	63.12	66.12	59.00	68.54	73.08	67.19
Unique alignment rate (%)	45.08	45.33	44.57	47.24	47.97	47.19
Multiple alignment rate (%)	41.88	42.20	43.03	44.28	43.81	44.53
Chimera alignment rate (%)	1.76	1.53	1.32	1.16	1.34	1.14
Overall alignment rate (%)	88.71	89.06	88.92	92.68	93.11	92.86
